# 3D‐Printed Ion‐Conductive Hydrogels with Tunable Mechanical–Electrical Properties for Multimodal Sign Language Recognition

**DOI:** 10.1002/advs.202520586

**Published:** 2026-01-20

**Authors:** Quan Hu, Longya Xiao, Peiqi Zhang, Qiurui Zhang, Guangsen Liu, Xinglin Qin, Zhuhui Yin, Xian Li, Yuling Wang, Hongjie Jiang

**Affiliations:** ^1^ Shien‐Ming Wu School of Intelligent Engineering South China University of Technology Guangzhou China; ^2^ School of Mechatronic Engineering Guangdong Polytechnic Normal University Guangzhou China; ^3^ Department of Rehabilitation Medicine The Sixth Affiliated Hospital of Sun Yat‐Sen University Guangzhou China; ^4^ Guangdong Provincial Clinical Research Center For Rehabilitation Medicine Guangzhou China

**Keywords:** ion‐conductive hydrogels, low hysteresis, multimodal sensing, sign language recognition

## Abstract

Sign language recognition technology holds significant importance for eliminating communication barriers faced by the hearing‐impaired population. To address the limitations of current wearable sensors‒such as complex fabrication or materials incompatibility within an integrated system, this study designed a 3D printed ion‐conductive hydrogel with tunable electromechanical performances for versatile wearable sensing. The hydrogel is primarily based on a polyampholyte network interpenetrated with a polyacrylamide (PAAM) framework and synergistically integrated with LiCl and a covalent organic framework (COF) to enhance its electromechanical performance. It exhibits low hysteresis (90.25% recovery ratio) with high elongation (550%) and large compressive strain tolerance (90%) for strain/pressure sensing, while its low modulus (0.09 MPa) and high conductivity (0.23 S m^−^^1^) enabled high‐fidelity surface electromyography (sEMG) sensing. Leveraging these multifunctional hydrogels, we developed a multimodal sign language recognition system consisting of a pair of digital gloves, each embedded with 12 strain sensors and 5 pressure sensors, together with a flexible armband integrated with a 10‐channel differential sEMG electrode array. Coupled with a bidirectional long short‐term memory (Bi‐LSTM) multimodal fusion model, the system achieved a classification accuracy of 99.65% across 24 Chinese sign language gestures.

## Graphical abstract

This work successfully fabricated ion‐conductive hydrogels with low hysteresis and high conductivity using 3D printing technology. By adjusting the component ratios, the properties of the hydrogels can be tuned to meet diverse sensing requirements. Finally, a multimodal sensing sign language recognition system was constructed based on this hydrogel, achieving accurate recognition of sign language.

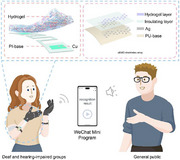

## 1 Introduction

Sign language serves as the primary communication method for deaf and hard‐of‐hearing individuals, playing a crucial role in their social integration [1]. Currently, over 1.5 billion people worldwide experience hearing loss, with projections indicating that this number will rise to approximately 2.5 billion by 2050 [2]. Professional sign language interpreters are costly and scarce, making the development of efficient sign language recognition systems a vital approach to breaking down communication barriers between hearing and non‐hearing populations. In recent years, rapid advancements in artificial intelligence and flexible electronics technologies [3, 4, 5, 6, 7]. have driven significant progress in sign language recognition methods based on computer vision [8, 9] and wearable sensors [10, 11]. Computer vision‐based approaches, however, demand high image quality and are susceptible to interference from occlusions and lighting variations, limiting their recognition performance in real‐world settings [12, 13]. In contrast, wearable sensors offer a more feasible solution for practical sign language recognition systems due to their compact size and continuous, precise motion capture capabilities [14, 15]. Currently, wearable sensors leveraging mechanisms such as strain sensing [16, 17], triboelectric effects [18, 19], and sEMG [20, 21]. have been integrated with artificial intelligence techniques to achieve accurate sign language recognition. For instance, Ma et al. developed a strain sensor (GF = 3.86) by encapsulating liquid metal particles within a hydrogel. Placed on the metacarpal bones of all five fingers, it monitored joint movements to recognize ten single‐hand sign language gestures [22]. Wen et al. developed a digital glove based on triboelectric sensors, with ten sensors placed on the metacarpal bones of all fingers, plus one sensor each on the fingertips of the right hand's index and middle fingers and the left hand's palm. Combined with a segmented convolutional neural network (CNN), it achieved 91.3% accuracy for 50‐word recognition and 95% accuracy for 20‐sentence recognition [23]. Moin et al. constructed a 64‐channel high‐density sEMG array using silver electrodes attached to the forearm. Combined with hyperdimensional computation, it achieved a 95.04% classification accuracy for 21 single‐handed gestures [24]. However, most existing sensors are primarily limited to detecting finger bending, making it challenging to simultaneously capture multimodal gesture features such as fingertip touch, palm interaction, and arm movement. This limitation leads to the omission of critical sign language components, thereby significantly restricting the recognition scope and practical application. To address this challenge, there is an urgent need to develop flexible sensors with integrated multimodal sensing capabilities.

In response, recent studies have increasingly focused on implementing multimodal sensing strategies for sign language recognition. For instance, Faisal et al. developed a data glove integrating five flexible strain sensors and an inertial measurement unit (IMU). The strain sensors were placed on the metacarpal regions of the five fingers on the right hand to capture finger bending, while the IMU was positioned on the dorsal side of the hand to monitor hand motion direction. By coupling this multimodal sensor system with a CNN, the model achieved a recognition accuracy of 82.19% for 24 static sign language gestures and 97.35% for 16 dynamic gestures [25]. Zhang et al. attached an 8‐channel sEMG electrodes to the right forearm and placed 10 IMUs on the five fingers (two IMUs per finger). Using a single‐camera multimodal fusion framework coupling with statistical and contrastive attention, this system achieved a recognition accuracy of 93.17% for 200 sign language words [26]. However, these sensors are composed of heterogeneous materials and lack intrinsic consistency in structural design and sensing performance. In contrast, hydrogels offer highly tunable electromechanical properties and mechanical compliance, making them promising candidates for integrated, engineerable multimodal sensors [27, 28, 29, 30, 31, 32, 33, 34]. From an electrical perspective, hydrogels used for multimodal wearable sensing can be classified according to the origin of charge carriers within the polymer network. Polyelectrolyte hydrogels, such as polyacrylic acid (PAA), partially dissociate in aqueous environments, resulting in pH‐dependent ionic transport. In contrast, neutral hydrogels, including poly(vinyl alcohol) (PVA) and PAAM, rely entirely on externally added electrolytes for ionic conductivity [35, 36, 37]. Gelatin behaves as an amphoteric hydrogel with pH‐ and temperature‐dependent charge states, whereas PDMAEA‐Q/PNaSS represents a strong polyampholyte system featuring permanently charged ionic groups and intrinsically stable ionic transport. Based on their sensing strategies, hydrogel‐based systems can be broadly categorized into the following types: i) Engineering distinct hydrogel formulations by adjusting monomer composition or cross‐linking density to respond to different physiological signals with the same type of electrical output, such as voltage or resistance [35, 36, 37, 38, 39]; ii) Incorporating more complex device geometries to enhance multimodal sensing and signal differentiation through geometric design, directing different stimuli to specific structural units; [40, 41] iii) Achieving signal‐diversified perceptions through intrinsic decoding of various external stimuli based on signal diversity, utilizing different types of electrical signals (e.g., voltage, current, or resistance changes) or variations in signal characteristics (e.g., distinct time response patterns) [42, 43, 44, 45, 46]. However, conventional hydrogels often suffer from significant hysteresis and residual strain under cyclic loading, which leads to signal drift, waveform distortion [47, 48, 49], and progressive degradation in mechanical and electrical performance due to moisture evaporation [50, 51, 52]. Consequently, it is imperative to engineer structurally customizable conductive hydrogels that simultaneously exhibit low hysteresis, high water retention, and multimodal sensing capabilities to advance the practical implementation of a sign language recognition system.

**FIGURE 1:**
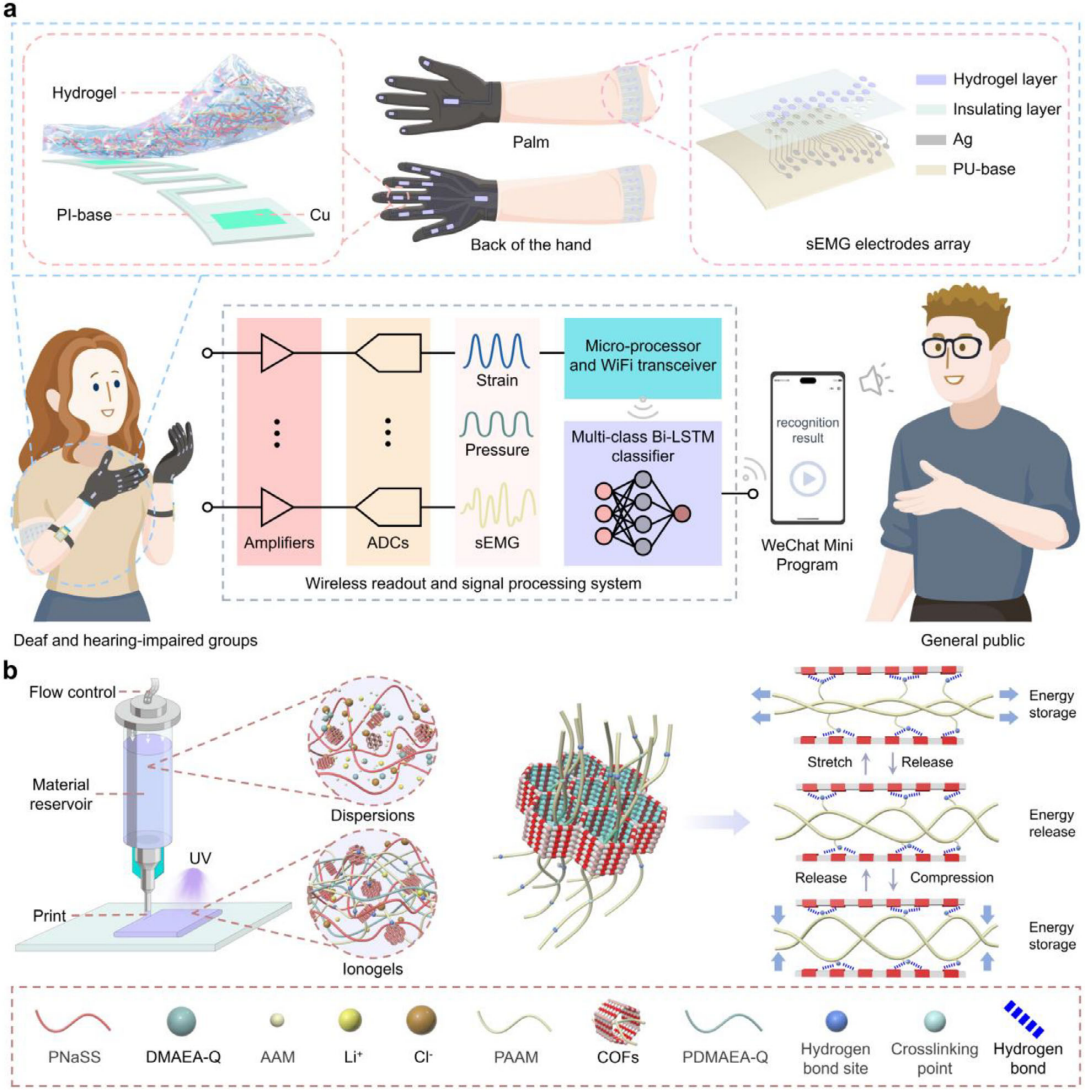
(a) Schematic illustration of the sign‐language recognition system integrating 12 strain sensors, 5 pressure sensors, and a 10‐channel differential sEMG electrode array, all derived from tunable ion‐conductive PIG. (b) Schematic illustration of 3D printing the PIG ink into a solid gel via UV polymerization, depicting the hierarchical crosslinking process that enables excellent hysteresis resistance and high mechanical resilience.

This work introduces a 3D‐printable, ion‐conductive hydrogel (PIG) with multimodal sensing capability, and further establishes a sign language recognition system based on this material (Figure 1a). PIG is engineered through a hybrid cross‐linking network, integrating a polyampholyte network with a covalently cross‐linked PAAM framework (Figure 1b). Within this framework, the carbonyl and amide groups of PAAM form strong hydrogen bonds with hydroxyl, carbonyl, and imine groups in COF (structure shown in Figure S1). These robust interfacial interactions suppress irreversible energy dissipation from polymer chain slippage under external forces, thus endowing PIG with low hysteresis. Moreover, by modulating cross‐linking density, polymer chain fraction, and gel charge density, the fracture elongation, modulus, and conductivity of the hydrogel can be tuned to accommodate diverse sensing requirements. Notably, PIG with 0.075 wt.% N,N'‐Methylenebisacrylamide (MBAA) and 5 mol/L acrylamide (AAM), designated as PIG_0.075‐5_, demonstrates exceptional mechanical properties, characterized by low hysteresis (elastic recovery rate of 90.25% at 300% tensile strain), high robustness (energy dissipation of 21.88 kJ m^−3^ at 300% tensile strain), and high extensibility (failure tensile strain of 550%). These features enable its effective application in strain and pressure sensing. In parallel, PIG_0‐5_ (0 wt.% MBAA and 5 mol/L AAM) exhibits a low elastic modulus of 0.09 MPa, high ionic conductivity of 0.23 S m^−1^, and low contact impedance of 3.39 kΩ (at 1 kHz), leading to outstanding performance in sEMG signal acquisition. When configured into flexible strain and pressure sensors, the hydrogel achieved a gauge factor of 2.3 with a linear strain response from 0%–550% and a highly linear pressure sensitivity of −0.013 kPa^−^^1^ over the 0–50 kPa range. Integrating 12 strain sensors and 5 pressure sensors onto a glove, combined with an sEMG electrode array and artificial intelligence algorithm, enabled the development of a multimodal sign language recognition system. The system successfully captured four categories of hand actions in real time, including finger bending, wrist movement, fingertip touch, and palm interaction. Processed by a Bi‐LSTM neural network, the multimodal sensor signals were transmitted to a WeChat program on a mobile device, achieving real‐time recognition of 24 common sign language gestures with an accuracy of 99.65%.

## 2 Results

### 2.1 3D Printing Properties of PIG

**FIGURE 2:**
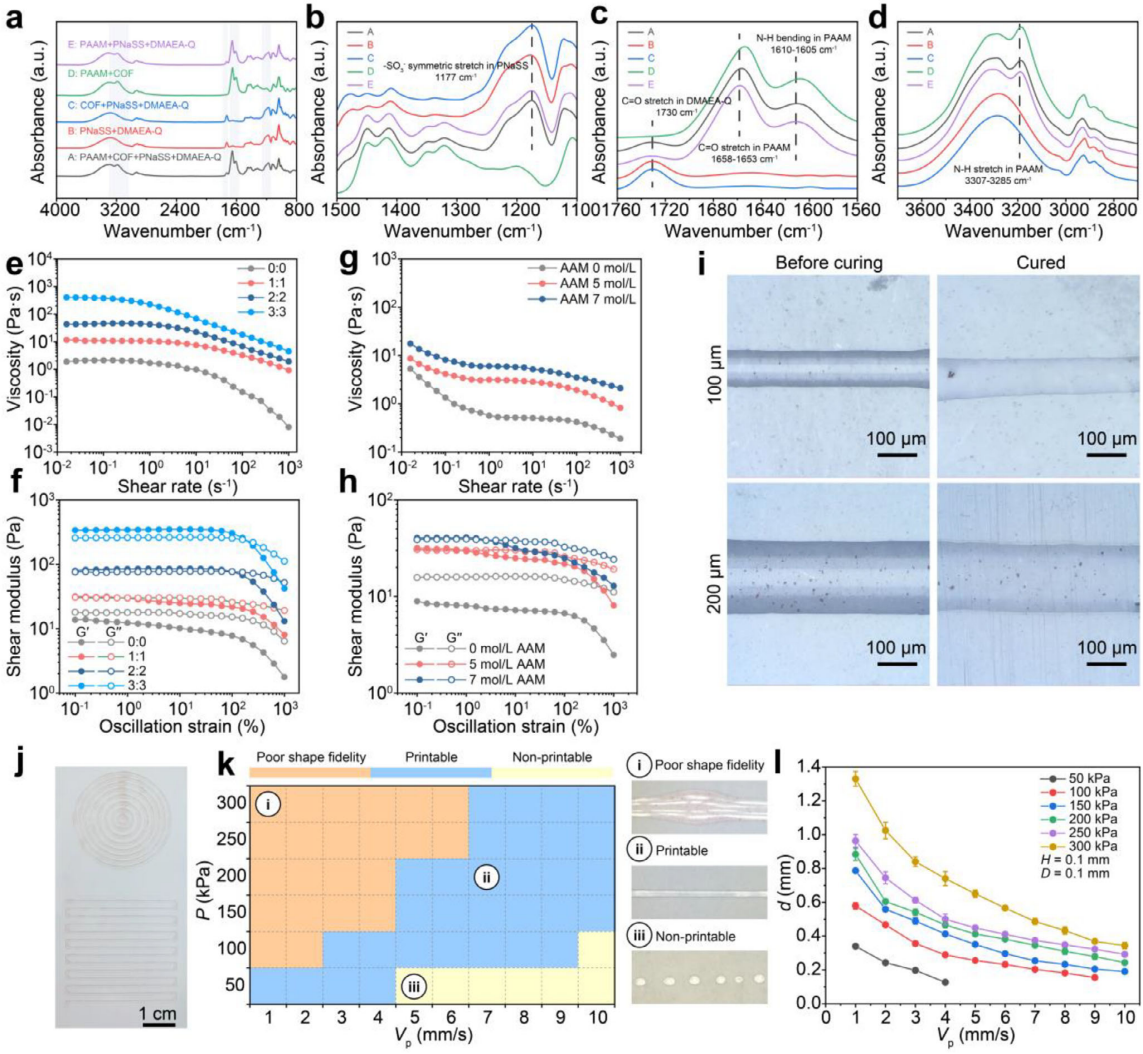
Spectroscopic characterization and rheological properties of PIG. (a–d) FT‐IR spectra confirming the molecular structure of PIG. (e, g)Viscosity and (f, h) *G* *′* and *G* *″* of PIG ink as functions of shear rate and oscillatory strain, respectively, under varying AAM concentrations (0–7 mol L^−^^1^) and PNaSS‐to‐DMAEA‐Q charge ratios (0:0–3:3). (i) Photographs of 3D‐printed PIG filaments extruded through nozzles with varying inner diameters before and after curing, and (j) printed PIG patterns demonstrating geometric fidelity. (k) Printability of PIG ink different applied pressures and printing speeds. (l) Relationship between filament diameter and printing velocity at different extrusion pressures.

The fabrication procedure for PIG is illustrated in Figure 1b. Initially, a COF, [2‐(dimethylamino) ethyl acrylate]‐quaternized (DMAEA‐Q), acrylamide (AAM), N,N'‐Methylenebisacrylamide (MBAA), glycerin, and LiCl were added to a pre‐synthesized poly(styrene sodium sulfonate) (PNaSS) solution. After stirring and ultrasonic dispersion, a homogeneous precursor solution was obtained. The desired gel geometry was 3D printed in the presence of the photoinitiator lithium phenyl (2,4,6‐trimethylbenzoyl) phosphate (Lap), and the cross‐linking reaction was initiated under UV irradiation. To investigate the interactions among the components in the synthesized hydrogel, its chemical structure was first characterized using Fourier transform infrared spectroscopy (FTIR, scanning range 400–4000 cm^−^^1^). Figure 2a–d displays the FT‐IR spectra of five different gel formulations, including A: PAAM‐COF‐PNaSS‐DMAEA‐Q; B: PNaSS‐DMAEA‐Q; C: COF‐PNaSS‐DMAEA‐Q; D: PAAM‐COF; E: PAAM‐PNaSS‐DMAEA‐Q. Comparing the spectra of A, D, and E, the characteristic absorption peak for the carbonyl (C═O) stretching vibration on the PAAM chain shifted from 1658 to 1653 cm^−^^1^ in both the A and D systems after COF introduction. Similarly, the characteristic absorption peak for the amino (N─H) bending vibration shifted from 1610 to 1605 cm^−^^1^. The N─H stretching vibration absorption peak also exhibited a significant red shift, moving from 3307 to 3285 cm^−^^1^. This red shift confirms the formation of strong hydrogen bonding interactions between the C═O and N─H groups on the PAAM molecular chain and the corresponding sites on the COF [47]. In contrast, the absorption peak positions of the sulfonate group (‐SO_3_^−^) in PNaSS and the carbonyl group (C═O) in DMAEA‐Q showed no significant shift upon COF introduction, indicating no effective interfacial interactions between COF and the PNaSS/DMAEA‐Q polyelectrolyte pair. Furthermore, X‐ray photoelectron spectroscopy (XPS) and Raman spectroscopy (600–3600 cm^−^^1^) results (Figures S2 and S3) further confirmed the successful synthesis of PIG.

The effects of polyelectrolyte charge density and monomer concentrations on the rheological properties of PIG ink were subsequently evaluated by adjusting the charge ratio of DMAEA‐Q to PNaSS and the concentration of AAM. For instance, at a charge ratio of 1:1, the total ion monomer concentration (*C*_M_) equaled to 1.15 mol L^−^^1^. When the charge ratio increased to 3:3, *C*_M_ correspondingly rose to 3.45 mol L^−^^1^. Rheological measurements (Figure 2e–h) indicate that inks lacking either DMAEA‐Q/PNaSS or AAM exhibited liquid‐like behavior, with the loss modulus (*G″*) consistently exceeding the storage modulus (*G′*) across the entire tested strain range. In contrast, PIG inks with a 1:1 charge ratio or containing 5 mol L^−^^1^AAM exhibited pronounced elastic behavior by *G* *′* > *G* *″*. Further increasing the charge ratio to 3:3 or the AAM concentration to 7 mol L^−^^1^ resulted in a substantial increase in viscosity, storage modulus, and loss modulus. This behavior is attributed to the formation of a dynamic ionic crosslinking network between quaternary ammonium cations in DMAEA‐Q and sulfonate ions in PNaSS, which significantly enhances the viscoelasticity of the precursor ink. Meanwhile, strong hydrogen bonding interactions between AAM and the COF further restrict polymer chain motion, leading to increased viscosity and pronounced shear‐thinning behavior.

Moreover, the ink's viscoelastic behavior demonstrated significant strain dependence. For instance, at oscillatory strain levels below 0.63%, the PIG ink with 5 mol L^−1^ AAM maintained elastic‐dominated behavior (*G* *′* > *G* *″*). However, beyond this threshold, *G* *′* and *G* *″* intersected, marking the onset of a viscoelastic transition. At 100% oscillatory strain, *G*″ substantially surpassed *G* *′*, and the ink transitioned into a quasi‐liquid state. This strain‐dependent viscoelasticity was further corroborated by the loss factor *δ* (*δ* = *G* *″*/*G* *′*), as shown in Figure S4a,b. Additionally, alternating strain trials were conducted on PIG inks with varying charge ratios to analyze their shear recovery behavior. As shown in Figure S4c,d, the shear recovery capability remained nearly constant at approximately 98% as the charge ratio increased from 0:0 to 2:2, but decreased markedly to 89% when the charge ratio is further increased to 3:3. This trend indicates that excessive charge density leads to higher effective cross‐linking density, which in turn impairs the dynamic recovery capability of the network. Based on the optimized rheological properties described above, the formulated gel was successfully printed into a hemispheric structure using a commercial inkjet printer, demonstrating its excellent printability (Figure S5a,b). Unless otherwise specified in subsequent experiments, the charge ratio between PNaSS and DMAEA‐Q is assumed to be 1:1.

The influence of printing parameters on the morphology of printed gel filaments was systematically investigated by varying the air pressure (*P*) and nozzle velocity (*V*_p_), while maintaining a constant nozzle height (*H* = 0.1 mm) and inner diameter (*D* = 0.1 mm). By carefully tuning these parameters, the gel could be printed into distinct patterns with negligible differences before and after curing (Figure 2i,j). The printability was assessed by measuring filament diameters using electron microscopy under varying nozzle velocity (1–10 mm s^−1^) and six different air pressure levels (50, 100, 150, 200, 250, and 300 kPa). As shown in Figure 2k, at low pressure and high velocity (e.g., 50 kPa and 5 mm s^−1^), filament breakage and discontinuity were observed. In contrast, at high pressure and low velocity (e.g., 300 kPa and 1 mm s^−1^), filament expansion was evident. This is because high *V*_p_ reduces the residence time in the nozzle, leading to stretching and thinning of the filament. Meanwhile, elevated *P* increases the extrusion rate, further intensifying filament expansion. The quantitative analysis of PIG ink printability was shown in Figure 2l. At constant pressure, the filament diameter negatively correlated with increasing *V*_p_. Conversely, at constant nozzle velocity, the filament diameter increases with increasing *P*, which is consistent with previous studies on extrusion‐based printing [34]. To validate ink uniformity, PIG samples with varying printed lengths (1–8 cm) were fabricated. A highly linear relationship was observed between printed length and electrical resistance, which increased from 113.2 to 912.5 kΩ (Figure S5c,d).

### 2.2 Mechanical Characterizations of PIG

**FIGURE 3:**
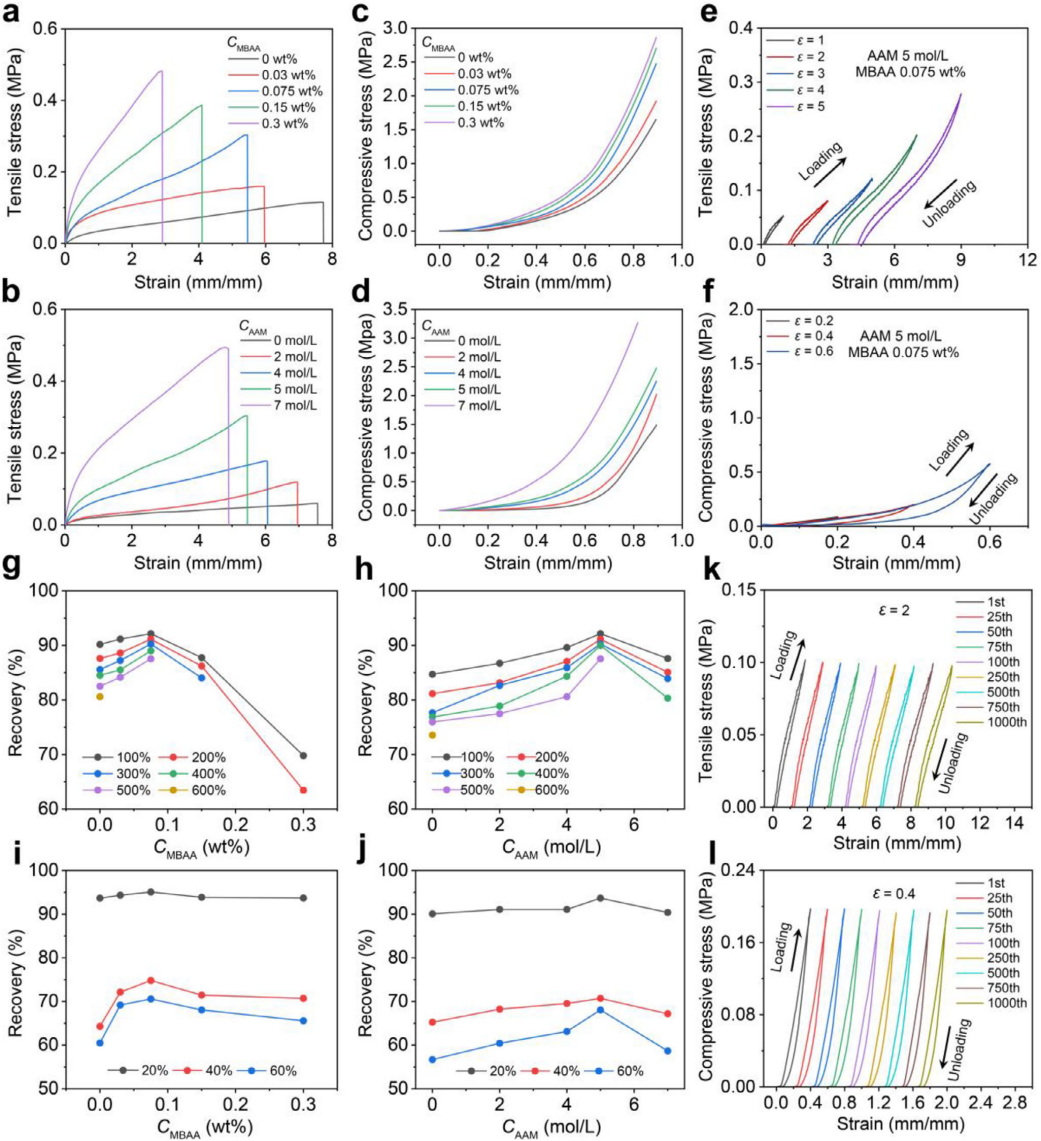
Mechanical characterizations of PIG. (a, b) Tensile stress–strain curves of PIG with varying MBAA and AAM contents, showing a linear increase in tensile strength and modulus (but not toughness) with increasing crosslinker and monomer content. (c, d) Compressive stress–strain curves under different MBAA and AAM concentrations, exhibiting similarly enhanced compressive properties. (e, f) Loading‐unloading stress–strain curves of PIG_0.075‐5_ under different tensile and compressive strain, respectively. (g, h) Recovery rate of PIG as a function of MBAA or AAM content under different tensile and (i, j) compressive strains, revealing the configuration of PIG_0.075‐5_ able to achieve the highest recovery ratio for both scenarios. (k, l) Cyclic loading‐unloading curves of PIG_0.075‐5_ over 100 consecutive cycles at 200% tensile strain and 40% compressive strain; horizontally offset curves correspond to the first, 25th, 50th, 75th, and 100th cycles.

The mechanical properties of PIG were systematically investigated by varying the composition ratio of MBAA or AAM. As shown in Figure 3a,b (with detailed data in Figure S6a,b), the tensile stress–strain curves demonstrated that both the Young's modulus (*E*) and fracture strength (*σ*_b_) increased significantly, e.g., from 0.09 to 0.96 MPa and from 0.0115 to 0.482 MPa, respectively, when the MBAA content was increased from 0 to 0.3 wt.% at a constant AAM concentration of 5 mol L^−1^. In contrast, the fracture elongation (*ε*_b_) decreased from 764% to 286%, indicating reduced stretchability. Interestingly, the tensile work (*W*_t_) displayed a non‐monotonic behavior: it initially increased from 0.4 MJ m^−^^3^ to a peak of 0.98 MJ m^−^^3^ as the MBAA content increased, and then stabilized around 0.9 MJ m^−^^3^. This suggests that a moderate degree of MBAA optimizes energy dissipation and toughness. However, excessive cross‐linking, while enhancing modulus and strength, restricts polymer chain mobility and limits further improvement in mechanical toughness. When varying AAM concentration at a fixed MBAA ratio of 0.075 wt.%, PIG demonstrated similar patterns in their Young's modulus and fracture strength, both of which increased with AAM concentration, whereas the fracture elongation decreased accordingly. Notably, the tensile work in this case showed a continuous upward trend, in contrast to the plateau observed with increasing MBAA. This difference can be attributed to the increase in polymer chain density due to higher AAM content, which improves the gel's load‐bearing capacity and enhances its ability to buffer deformation and dissipate mechanical energy, ultimately leading to improved toughness and extensibility.

The compressive properties of PIG were evaluated using the same experimental approach as the tensile tests. Specifically, either the MBAA content was varied while maintaining the AAM concentration at 5 mol L^−1^, or the AAM concentration was varied while fixing the MBAA content at 0.075 wt.%. The corresponding results are presented in Figure 3c,d and Figure S6c,d. Upon a large compressive strain of 80%, all compressive parameters, including compressive strength, compressive modulus, and compressive work, demonstrated a linearly positive correlation with both MBAA and AAM content. Similar to the observed enhancement in tensile properties, the improvement in compressive performance is attributed to increased crosslinking density with higher MBAA content, and to elevated polymer chain density with higher AAM concentration. These structural reinforcements enable the hydrogel to sustain higher mechanical loads and better resist deformation (e.g., a compressive strain of 90%).

To reduce the mechanical hysteresis of PIG, COF was added to the hydrogel formulation. The addition of COF nanosheets introduces rigid, porous structures that act as physical crosslinks and anchoring points, enhancing network elasticity and reducing internal friction. Their high surface area enables more uniform stress distribution and minimizes localized strain concentration. Together with the abundant hydrogen‐bonding interactions formed with AAM, this structure promotes reversible deformation of the PIG network, thereby enhancing its hysteresis resistance. The effect of COF incorporation on reducing the mechanical hysteresis of PIG was first evaluated by varying the content of either MBAA or AAM, as presented in Figure S7 and Figure 3e,f. As the content of MBAA or AAM increased, the area of the mechanical hysteresis loop during cyclic deformation was observed to initially decrease and then increase. These phenomena were observed consistently in both tensile and compressive tests. The hysteresis resistance of PIG was further evaluated by calculating the recovery ratio (*W*_unloading_/*W*_loading_), defined as the ratio of the integrated areas under the loading and unloading stress–strain curves. As shown in Figure 3g–j and Figure S8, across tensile strains from 100% to 600% and compressive strains from 20% to 60%, the recovery rate of all PIG formulations exhibited a trend of initially increasing and then decreasing with increasing MBAA or AAM content. Among them, PIG_0.075‐5_ (0.075 wt.% MBAA and 5 mol L^‒1^ AAM) achieved the highest recovery ratio under either tensile or compressive loading. For instance, at *ε* = 3 (tensile) and *ε* = 0.6 (compressive), the recovery rates of PIG_0.075‐5_ were 90.25% and 70.57%, respectively. This non‐monotonic trend arises from the interplay between energy dissipation and structural elasticity within the gel network. At low concentrations, the polymer network is loosely cross‐linked or sparsely populated, leading to significant chain slippage and internal friction, thereby generating higher hysteresis. As the content of MBAA or AAM increases, the cross‐linking density and polymer backbone density are enhanced, which improves structural recovery and reduces energy loss during cyclic deformation. However, excessive cross‐linking (via MBAA) or over‐densification (via AAM) restricts polymer chain mobility and hampers network relaxation during unloading. This leads to internal stress accumulation and irreversible deformation, which in turn enlarges the hysteresis area again.

The dynamic hysteresis resistance of PIG_0.075‐5_ was evaluated under cyclic loading‐unloading for 1000 cycles at varying tensile and compressive strains, with recovery ratios compared between cycle 1 and 1000. As shown in Figure S9, across tensile strains from 100% to 300% and compressive strains from 20% to 60%, PIG_0.075‐5_ exhibited negligible differences after 1000 cycles. Specifically, at tensile strain of *ε* = 2 and compressive strain of *ε* = 0.4, the recovery ratios were 80.61% and 63.45% after 1000 cycles, compared to 91.12% and 74.81% before cycling (Figure 3k,l). Moreover, residual strains remained low (0.45 and 0.093, respectively). These results confirm the excellent mechanical recoverability of PIG_0.075‐5_.

**FIGURE 4:**
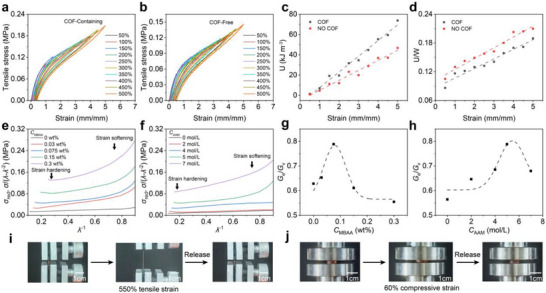
Energy dissipation analysis of PIG. (a, b) Continuous cyclic tensile loading‐unloading stress–strain curves of PIG_0.075‐5_ with/without COF. (c, d) Relationship curves of dissipated energy (U) and the ratio of dissipated energy to tensile work (U/W) versus strain for PIG_0.075‐5_ with/without COF. (e, f) Residual stress (*σ*_red_) versus *λ* ^−1^ curves for PIG. (g, h) *G*_v_/*G*_e_ ratio versus MBAA or AAM content after fitting the tensile stress–strain curves of PIG. (i, j) Photographs of PIG_0.075‐5_ before and after 500% elongation and 60% compression, respectively.

To investigate the influence of COF on the energy dissipation behavior of PIG, PIG_0.075‐5_ and its COF‐free control sample was subjected to continuous cyclic tensile loading–unloading tests with gradually increasing strain values (50%–500%) and no dwell time (Figure 4a,b). The stress–strain hysteresis loops of the COF‐containing samples exhibited a higher degree of overlap between adjacent cycles, indicating superior elastic recovery. Quantitative analysis was performed by calculating the dissipated energy (U) at different strain levels and the ratio of dissipated energy to total tensile work (U/W). Here, U represents the energy dissipated during a complete loading‐unloading cycle, while U/W denotes the fraction of irreversible tensile work associated with internal network damage. A higher U/W value indicates more severe structural disruption of the gel network. As shown in Figure 4c,d, the introduction of COF significantly increased the U value of PIG_0.075‐5_ while simultaneously reducing its U/W ratio. For example, at *ε* = 5, U increased from 46.74 to 73.79 kJ m^−^^3^, while U/W decreased from 20.98% to 18.94%. This indicates that COF enhances energy dissipation efficiency while suppressing irreversible network damage, thereby reducing hysteresis. This behavior is consistent with the results obtained from continuous cyclic compression tests (Figure S10), confirming that COF incorporation effectively improves fatigue resistance and rapid recovery capability while simultaneously enhancing the mechanical robustness of the PIG network.

The influence of polyelectrolyte charge density on the energy dissipation behavior of PIG_0.075‐5_ was investigated through continuous cyclic tensile tests with varying PNaSS:DMAEA‐Q charge ratios from 0:0 to 3:3 (Figure S11). The results indicate that both U and U/W significantly increased with rising total charge. This phenomenon stems from the enhanced ionic crosslinking density, which increases the rigidity of the polymer network and restricts the motion of molecular segments. The resulting stiffened network promotes stronger internal friction and irreversible plastic deformation during cyclic deformation, thereby elevating the level of energy dissipation while intensifying the hysteretic behavior.

The nonlinear elastic behavior of PIG was analyzed using the phenomenological Mooney‐Rivlin equation [53, 54], which captures the apparent strain softening and hardening behavior under large deformation:

(1) $$\sigma_{\mathrm{red}}=\frac{\sigma }{\lambda -\lambda^{-2}}=2C_{1}+2C_{2}\frac{1}{\lambda }$$

where *σ*_red_ denotes reduced stress, *λ* represents the elongation ratio (*λ* = *ε* + 1), and *C*_1_ and *C*_2_ are material constants. 2*C*_1_ equals the shear modulus (≈*E*/3), while *C*_2_ correlates with strain hardening (*C*_2_ < 0) and/or strain softening (*C*_2_ > 0) beyond the Gaussian elastic region. *C*_2_ = 0 indicates the material is in the purely elastic tensile region. In PIG, strain softening is primarily attributed to the breaking of sacrificial hydrogen bonds, while strain hardening originates from quasi‐permanent cross‐links (e.g., tough dynamic bonds) or chemical cross‐links [54, 55, 56]. We plot *σ*_red_ versus *λ* ^−^^1^ relationship curves for PIG hydrogels with different compositions (Figure 4e,f). The resulting curves revealed a composition‐dependent nonlinear elastic response. Specifically, PIG_0.075‐5_ exhibits an apparent strain‐softening regime at *λ* ^−^^1^ < 0.23, followed by a transition to strain‐hardening behavior when *λ* ^−^^1^ > 0.23.

To investigate the effects of MBAA and AAM concentrations on the viscoelasticity of PIG, we further employed the viscoelastic model proposed by Creton et al. (see the details in the Supporting Information) [54, 55, 56]. This model decomposes the material's initial shear modulus (*G*) into viscoelastic (*G*_v_) and elastic (*G*_e_) components and has been successfully applied to analyze the nonlinear mechanical behavior of materials such as polyacrylic acid gels [54, 55, 56]. Figure S12 presents representative stress–strain curves alongside their simulated counterparts based on this model. Fitting was performed using tensile data from Figure 3a,b (initial strain rate $\dot{\varepsilon }$ = 0.14 s^−^^1^), with results shown in Figure 4g,h, and Figure S13. Both *G*_v_ and *G*_e_ exhibited increasing trends with rising MBAA and AAM concentrations, indicating their synergistic enhancement of the hydrogel network structure. Notably, the *G*_e_/*G*_v_ ratio‐a key parameter characterizing material elasticity‐exhibited an initial increase followed by a decrease, peaking at an MBAA concentration of 0.075 wt.% and an AAM concentration of 5 mol L^−1^. This result aligns with the recovery behavior of samples shown in Figure 3g,h, further demonstrating that the model effectively captures the tensile behavior of the gels in our work.

Considering overall performance, PIG_0.075‐5_ with a charge ratio of 1:1 was selected as the optimal formulation for both strain and pressure sensing, as it effectively minimized mechanical hysteresis while balancing mechanical strength and energy dissipation. It maintains excellent mechanical properties (*E* = 0.26 MPa, *ε*_b_ = 550%) and achieves a recovery rate of 90.25% and 70.57% as well as energy dissipation of 21.88 and 37.43 kJ m^−^^3^ at representative strain levels of ε = 300% (tensile) and 60% (compressive). It maintains structural stability even after large deformations, including 500% tensile strain and 60% compressive strain (Figure 4i,j). In addition, the tearing fracture energy (*T*) of PIG_0.075‐5_ was determined via trouser tearing tests (Figure S14), yielding a high tear energy of 752 J m^−2^.

### 2.3 Electrical and Multimodal Sensing Performances of PIG

Figure S15a,d presents the AC impedance of the hydrogel in the configuration of PIG as a function of excitation frequency. The impedance decreased with increasing frequency, a trend attributed to the resistance‐capacitance (RC) behavior at the hydrogel–electrode interface: higher frequency, lower impedance. EIS fitting analysis of Nyquist plots for PIG with different compositions revealed that the gel bulk resistance (*R*_e_) increased with rising MBAA or AAM content (Figure S15c,f and Table S1). This indicates that higher MBAA or AAM content reduces the gel's conductive ability. The maximum conductivity of the gel was 0.23 S m^−^^1^ at a configuration of 0% MBAA and 5 mol L^−1^ AAM (denoted as PIG_0‐5_) with charge ratio of PNaSS:DMAEA‐Q = 1:1 (Figure S16). Additionally, the conductivity of PIG_0‐5_ decreased as the total charge of PNaSS and DMAEA‐Q increased. This reduction can be explained by the denser polymer network formed at higher crosslinking or chain concentrations, which restricts ion migration and thus increases impedance.

**FIGURE 5:**
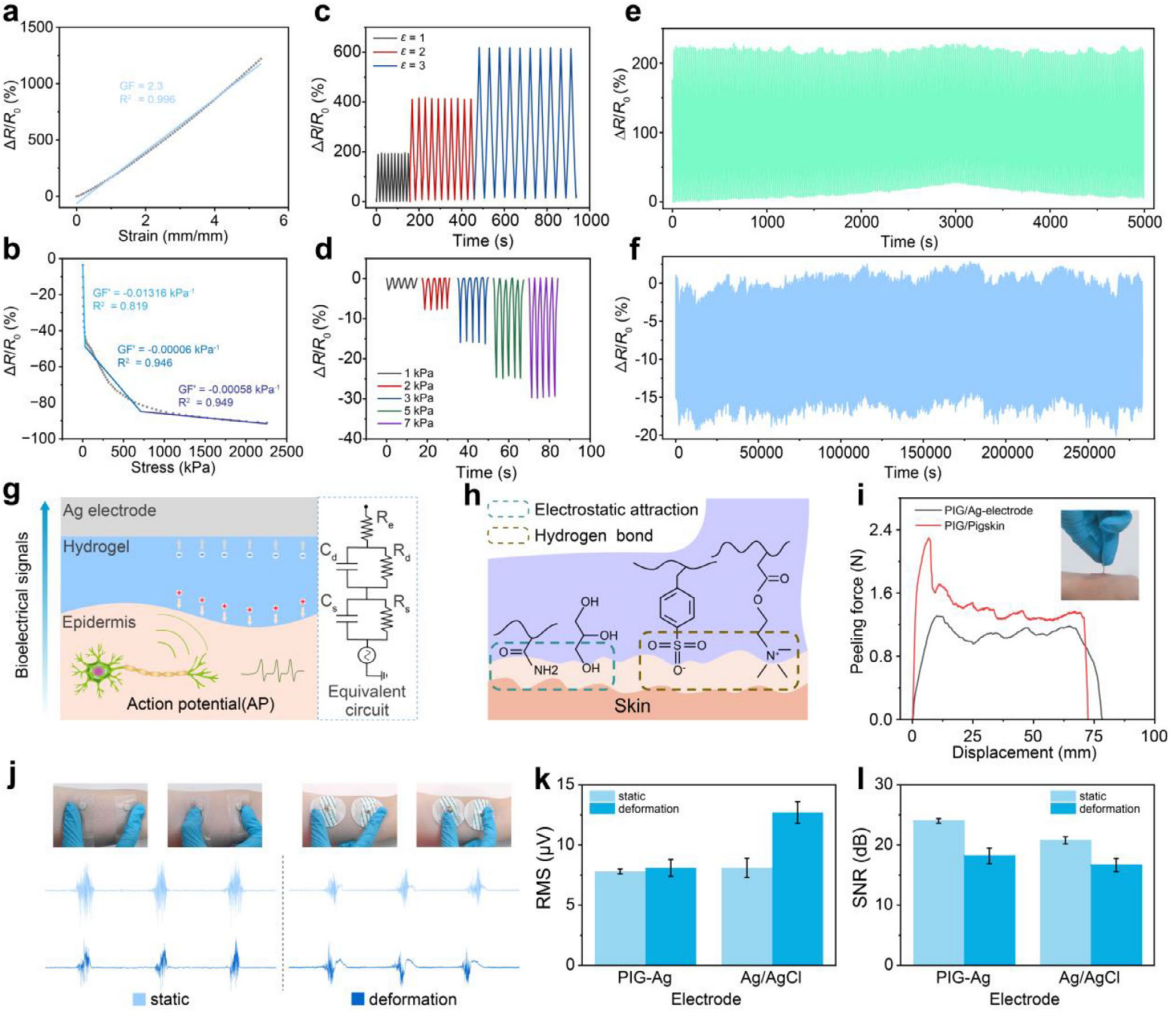
Sensing performance of PIG. (a) Relative resistance variation as a function of tensile strain, with the slope indicating the strain sensitivity. (b) Relative resistance variation as a function of compressive strain, with the slope indicating the pressure sensitivity. (c, d) Relative resistance changes of the PIG_0.075‐5_ under different tensile strains (c) and applied pressures (d). (e, f) Cyclic sensing stability of the PIG_0.075‐5_ after 500 tensile cycles (e) and 5000 compressive cycles (f). (g) Schematic illustration of electron‐ion conversion at the epidermis‐electrode and its equivalent RC circuit model. (h) Schematic of adhesion force generation mechanism in PIG arising from hydrogen bonding and electrostatic interactions. (i) Adhesion strength of PIG_0‐5_ on porcine skin and Ag electrode; inset shows a photograph of PIG_0‐5_ adhering to skin. (j–l) Comparison of sEMG signal quality between PIG_0‐5_‐Ag and commercial Ag/AgCl electrodes under cyclic deformation, demonstrating superior motion‐artifact resistance of PIG_0‐5_‐Ag electrodes.

As mentioned previously, the optimized configuration of PIG_0.075‐5_ was employed to construct strain and pressure sensors. When an external force is applied, the hydrogel network undergoes macroscopic deformation. Simultaneously, the migration pathways of free Li^+^/Cl^−^ ions within the confined spaces at crosslinking points change, ultimately manifesting as changes in electrical resistance [17]. The strain sensitivity was quantified by the gauge factor (GF), defined as GF = (Δ*R*/*R*_0_)/*ε*, where *ε* denotes tensile strain. Pressure sensitivity was expressed as GF' = (Δ*R*/*R*_0_)/*σ*, where *σ* represents compressive strain. As shown in Figure 5a,b, the PIG_0.075‐5_‐based strain sensor exhibited a highly linear sensitivity with a GF of 2.3 across the entire 0%–550% strain range. For pressure sensing, within the low‐pressure regime of 0–50 kPa, the sensor achieved a sensitivity of GF' = −0.013 kPa^−^^1^, enabling precise detection of subtle fingertip pressures. However, beyond 50 kPa, the pressure sensitivity decreased markedly.

Figure 5c,d illustrates the dynamic sensing behavior of the strain and pressure sensors under progressively increasing loads. For tensile testing, the PIG_0.075‐5_‐based strain sensor was subjected to cyclic loading‐unloading at strains of 100%, 200%, and 300%. For pressure sensing, the device was tested under pressures of 1, 2, 3, 5, and 7 kPa. In each case, the sensors were cycled for 10 (strain) or 5 (pressure) repetitions. The results revealed consistent resistance variations across repeated cycles, with relative resistance increasing proportionally with applied strain or pressure. To further assess durability, extended cyclic tests were conducted: the strain sensor was repeatedly stretched for 500 cycles at 200% strain, while the pressure sensor was compressed for 5000 cycles at 3 kPa. As shown in Figure 5e,f, no significant signal degradation was observed in either case, demonstrating excellent long‐term operational stability and reliability. Additionally, the cyclic long‐term stability of PIG was evaluated by examining the signal drift rate under cyclic tensile/compressive loading, with varying MBAA and AAM concentrations (Figure S17). PIG_0.075‐5_ exhibited the lowest drift rates, with a strain signal drift rate of 3.24% and a pressure signal drift rate of 7.05%.

Besides the strain and pressure sensors, the third sensing modality implemented in this work was a sEMG electrode array. Each electrode consisted of a PIG directly laminated onto the skin, with a silver (Ag) conductive trace deposited on the top surface of the gel, yielding a PIG‐Ag electrode. The corresponding equivalent circuit is presented in Figure 5g, where the recording quality of sEMG signals is governed primarily by the hydrogel–epidermis interface, modeled as an RC module. Unlike strain and pressure sensors, the sEMG electrodes were fabricated using PIG_0‐5_ (0 wt.% MBAA, 5 mol L^−1^ AAM, and Charge ratio of 1:1)_,_ a formulation with a lower modulus, thus offering superior compliance to skin tissue. Such compliance is critical because dynamic muscle contractions can induce micron‐scale interfacial gaps between the electrode and the deforming skin surface. This mechanical mismatch disrupts the electron‐ion capacitive coupling at the interface, leading to reduced interfacial charge density and increased contact impedance. Consequently, these effects introduce elevated noise levels and distort the acquired sEMG signals.

These challenges are effectively mitigated by the strong interfacial interactions formed between PIG_0‐5_ and skin. Such interactions arise from synergistic hydrogen bonding and electrostatic attractions enabled by the high ion density within the hydrogel network (Figure 5h). The interfacial adhesion was quantitatively evaluated using a 180° peel test. As shown in Figure 5i, PIG_0‐5_ exhibited strong adhesion strengths of 73.46 N m^−1^ to pig skin and 97.06 N m^‒1^ to Ag electrodes. Furthermore, the superiority of this formulation was corroborated by comparative contact impedance measurements between PIG electrodes and skin across different compositions. As shown in Figure S18a,b, the contact impedance increased monotonically with increasing MBAA and AAM content, confirming that lower crosslinking density and reduced modulus are essential for achieving low‐impedance, high‐fidelity sEMG recording. The collaboration between mechanical strengths and sEMG signal quality in PIG‐Ag electrodes was further evaluated by assessing their resistance to motion artifacts. A dynamic interference test was designed in which subjects performed a 50% maximum voluntary contraction (MVC) grip task while periodic tensile and compressive disturbances were applied to the skin at the electrode site. During this test, PIG compositions were systematically varied, and the resulting motion‐induced noise was quantified by analyzing the root‐mean‐square (RMS) value of the noise signal and the corresponding signal‐to‐noise ratio (SNR). As shown in Figure S18c,d, increasing the MBAA or AAM content led to a pronounced increase in RMS noise accompanied by a corresponding decrease in SNR. These results indicate that higher crosslinking density and modulus exacerbate motion‐induced interfacial instability. Collectively, these findings confirm that PIG‐Ag electrodes fabricated from PIG_0‐5_ exhibited superior adhesion, reduced motion artifacts, lower noise levels, and higher SNR, underscoring their suitability for stable, high‐fidelity sEMG recording during dynamic motion.

The motion‐artifact resistance of PIG_0‐5_‐Ag electrodes was systematically compared with that of commercial Ag/AgCl electrodes serving as controls. Under mechanical disturbance, the Ag/AgCl electrodes exhibited noticeable displacement from their original positions, whereas the PIG_0‐5_‐Ag electrodes maintained intimate contact with the skin (Figure 5j). Quantitative analysis of contraction‐induced baseline noise was performed by calculating the RMS values of noise level and SNR. As shown in Figure 5k, the PIG_0‐5_‐Ag electrodes consistently exhibited lower RMS noise compared to Ag/AgCl electrodes. Under static conditions, the PIG_0‐5_‐Ag electrode exhibited an RMS of 7.8 µV, slightly lower than the Ag/AgCl electrode (8.1 µV). During dynamic deformation, the PIG‐Ag electrode RMS increased marginally to 8.3 µV (6.4% increase), whereas the Ag/AgCl electrode RMS rose sharply to 12.7 µV (56.8% increase). Correspondingly, Figure 5l reveals that the PIG_0‐5_‐Ag electrode achieved a higher SNR of 23.99 dB (static) and 18.15 dB (dynamic), surpassing the Ag/AgCl values of 20.77 and 16.65 dB, respectively.

To evaluate the advantages of 3D‐printed PIG over cast PIG for multimodal sensing, their interfacial electromechanical performances were systematically compared. The adhesion strength between printed PIG_0.075‐5_ and Cu electrodes (for strain and pressure sensing) was significantly higher than that of the cast samples (Figure S19a). During 100 consecutive 90° bending cycles, the interfacial impedance of printed PIG_0.075‐5_‐Cu electrodes consistently remained lower than that of cast PIG across the entail excitation frequency from 10^−1^ to 10^6^ Hz (Figure S19b). A similar trend was observed for printed PIG_0‐5_‐Ag electrodes (for sEMG sensing), which also exhibited lower interfacial impedance than their cast counterparts (Figure S19c). During dynamic muscle contraction tests at 50% MVC, sEMG signals acquired using printed PIG_0‐5_‐Ag electrodes showed a SNR and lower noise RMS values compared with cast PIG electrodes (Figure S19d–f). Collectively, these results demonstrate that the 3D printing process enables the formation of a more robust and stable PIG–electrode interface than conventional fabrication approaches that rely on transferring prepolymerized hydrogels onto conductive electrodes, thereby significantly enhancing the reliability and signal quality of multimodal sensing acquisition.

### 2.4 Long‐Term Stability of PIG

**FIGURE 6:**
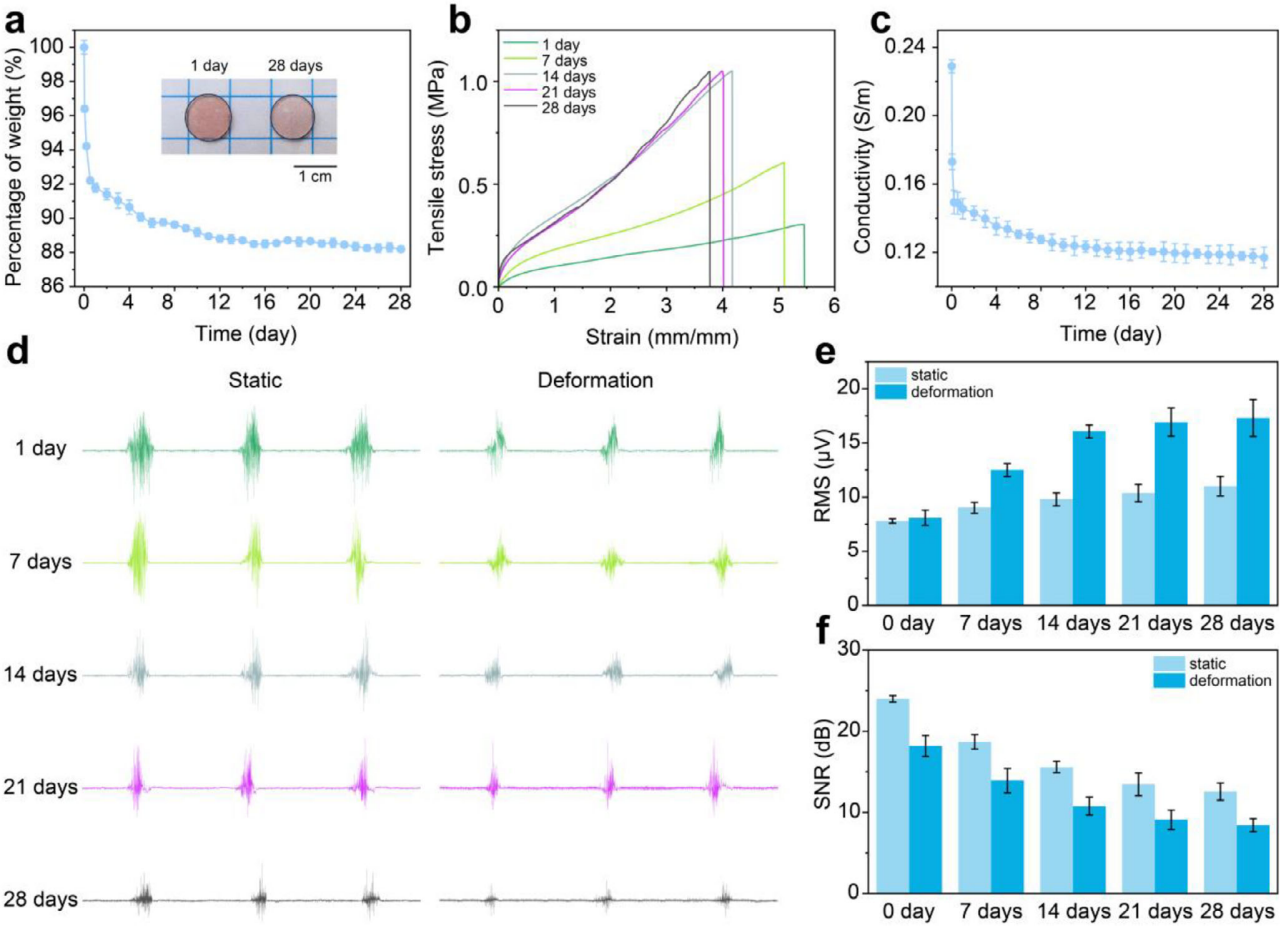
Long‐term stability of PIG. (a) Weight variations of PIG_0.075‐5_ over a 28‐day period under ambient conditions. (b) Evolution of tensile stress–strain curves for PIG_0.075‐5_ during 28 days of storage. (c) Change in electrical conductivity of PIG_0‐5_ over 28 days as a function of storage time. (d–f) Comparison of sEMG signal quality recorded by PIG_0‐5_‐Ag electrodes under cyclic deformation conditions after different placement durations.

To systematically evaluate the long‐term stability and reliability of PIG, a 28‐day anti‐dehydration test was performed under constant ambient conditions (25°C, 70% relative humidity). The water‐retention ability of PIG_0.075‐5_ was quantified by continuously monitoring weight variations over the test period. As shown in Figure 6a, the gel exhibited rapid weight loss during the first 168 h, stabilizing at 88.2% of its initial weight after 336 h (14 days), without noticeable changes in geometric dimensions. This stability was attributed to dual molecular mechanisms: hydrogen bonding between glycerol and water molecules that limits evaporation, and the hygroscopic nature of LiCl, which further suppresses moisture loss. The influence of dehydration on mechanical performance was characterized by tensile testing at different storage times (Figure 6b). Both fracture strength and Young's modulus increased monotonically with time, while fracture elongation decreased gradually, with all properties stabilizing after 14 days (Figure S20a). The behavior is attributed to water loss, which increases the volume fraction of polymer chains, reduces the average interchain spacing, and enhances molecular chain friction resistance. Consequently, the gels displayed higher strength, modulus, and tensile work, but reduced extensibility. Long‐term electrical stability was also assessed (Figure 6c). The conductivity of PIG_0‐5_ decreased progressively from 0.23 to 0.12 S m^−1^ over 28 days. This decline correlates directly with reduced water content, which impedes Li^+^/Cl^−^ ions mobility, leading to diminished charge transport capacity.

To further assess the stability of PIG_0‐5_ in electrophysiological applications, 50% MVC dynamic interference tests were conducted every 7 days over a 28‐days period. As shown in Figure 6d, on day 1 the RMS values of sEMG signals in static and dynamic deformation states were 7.8 and 8.3 µV, respectively, with corresponding SNRs of 23.99 and 18.15 dB. Over time, performance was observed to steadily decline and gradually stabilized after 14 days. The RMS values gradually increased, reaching 11 µV (resting) and 17.3 µV (dynamic) by day 28, while SNRs decreased to 12.6 and 8.4 dB, respectively (Figure 6e,f). This decline in recording quality can be attributed to dehydration‐driven changes in the hydrogel network. Specifically, increased modulus and reduced conductivity elevated the hydrogel‐skin interfacial impedance (Figure S20b), thereby reducing charge‐transfer efficiency. Despite these degradations at later stages, the PIG_0‐5‐_Ag electrode maintained stable sEMG signal quality during the first week, demonstrating reliable performance for short‐term wearable monitoring. Importantly, its initial stability and tunable interfacial adhesion highlighted the potential of PIG_0‐5_ for longer‐term bioelectronic interfaces, provided strategies to mitigate dehydration are incorporated.

### 2.5 Development of a Multimodal Sign Language Recognition System

**FIGURE 7:**
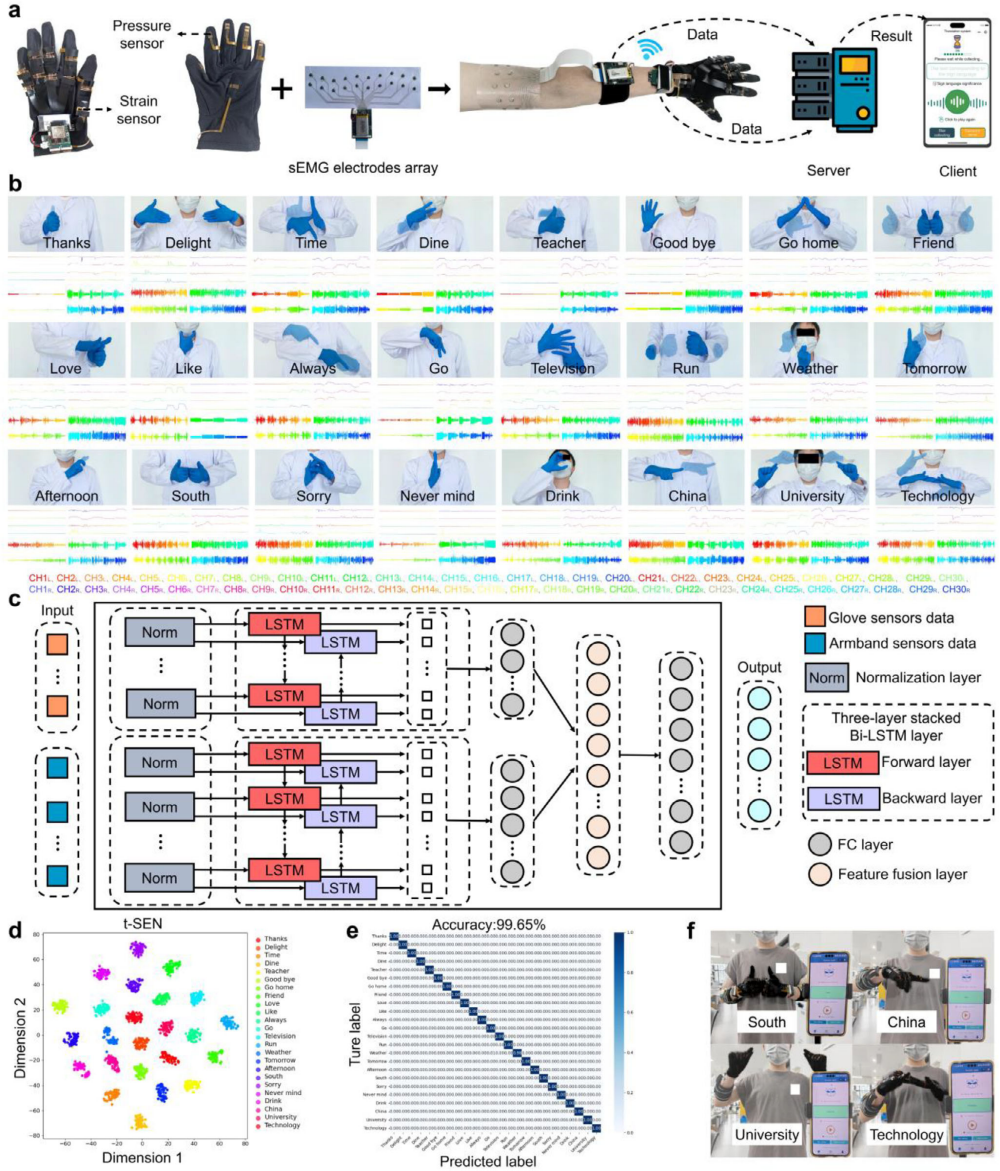
Schematic illustration of the intelligent sign language recognition system. (a) Principal components of the multimodal sign language recognition system, consisting of strain, pressure, and sEMG sensing. (b) Twenty‐four representative Chinese sign language gestures with corresponding multimodal sensing signals; semi‐transparent and opaque hand images denote the initial and final postures, respectively. (c) Architecture of the AI framework for multimodal sign language recognition based on a Bi‐LSTM fusion model. (d) Word‐level feature clustering derived from the Bi‐LSTM output layer. (e) Confusion matrix the classification results of 24 sign language gestures. (f) Demonstration of real‐time translation of the sign ‘South China University of Technology’ into text and speech on the mobile application interface.

Leveraging the multimodal sensing capabilities of PIG, we developed an intelligent sign language recognition system comprising a pair of digital gloves and a flexible armband, which collectively capture finger, palm, wrist, and muscle activities (Figure 7a). The acquired multimodal signals are wirelessly transmitted to a cloud server and converted into speech via a custom‐developed mobile application.

The gloves were fabricated by depositing PIG_0.075‐5_ onto predefined locations of palm‐patterned flexible printed circuit boards (FPCBs). To accommodate the repeated bending motions at finger joints, signal transmission traces were fabricated in serpentine geometries (Figure S21a,b). Each sensing glove system integrates 12 strain sensors (dimensions: 15 × 5 × 1 mm^3^) and 5 pressure sensors (dimensions: 5 × 5 × 1 mm), which were fabricated using 3D printed PIG_0.075‐5_. Specifically, two strain sensors were positioned at the proximal and distal interphalangeal joints of each finger, respectively, while a single pressure sensor was located at the fingertip. An additional strain sensor was mounted on the palm and another on the wrist, enabling comprehensive capture of all five finger articulations together with palm and wrist motions. To complement the multimodal sensing capabilities of the glove, a 10‐channel differential sEMG electrode array was fabricated through screen printing and layer‐by‐layer imprinting techniques. The array consists of 20 individual PIG–Ag composite electrodes, each comprising a 3D‐printed PIG_0‐5_ disk (10 mm diameter and 1 mm thick printed) integrated onto Ag electrodes. The complete electrode array was subsequently laminated onto a flexible armband for conformal skin contact (Figure S21c). This array was designed to record real‐time muscle activities from the upper arm, providing complementary neuromuscular signals to enhance gesture classification accuracy. Strain/pressure and sEMG acquisition were detailed in Figure S22. Sensor outputs were wirelessly transmitted via a Wi‐Fi module to a cloud server using the WebSocket protocol. On the server side, a pre‐trained multimodal fusion deep learning model was deployed to synchronously process the strain/pressure data and sEMG signals corresponding to sign language. Recognition results were displayed in real time as text on a WeChat mini‐program client, accompanied by concurrent playback of synthesized speech. This cross‐modal translation from sign language to spoken language establishes a closed‐loop human–machine interaction framework, enabling intuitive and practical communication for sign language users.

This study employed Chinese Sign Language (CSL) to construct a dataset of 24 commonly used everyday gestures. Representative sign images, together with their corresponding strain, pressure, and sEMG signals, are illustrated in Figure 7b. The strain and pressure sensors were sampled at 200 Hz, while sEMG signals were sampled at 1000 Hz. Each gesture consisted of 120 data samples, with each sample containing 2 s of multimodal time‐series data, yielding a total of 56 000 data points. Specifically, this dataset contained 400 × 40 dimensional strain/pressure data (containing 6 dummy channels) and 2000 × 20 dimensional sEMG data. To quantitatively assess signal similarity and correlation among the 24 sign categories, the mean feature vectors of the 120 samples were extracted as reference templates. Subsequently, Pearson correlation coefficients were calculated between new input signals and each template (Figure S23). The resulting correlation coefficient distribution revealed that multiple sign language signals fell within the strong correlation region, indicating significant similarity among these signs and a higher risk of misclassification.

The dataset was partitioned into training, validation, and test sets in an 8:1:1 ratio, coupling with a built deep learning algorithm to classify the 24 gestures. To address the heterogeneous nature of strain/pressure and sEMG signals, a multimodal fusion deep learning model was designed (Figure 7c). This model adopted a dual‐branch architecture, enabling independent processing of strain/pressure and sEMG signal streams. Each branch contained a three‐layer stacked Bi‐LSTM network followed with two fully connected (FC) layers, with outputs fused a feature integration layer. Specifically, the strain/pressure sensing branch employed a Bi‐LSTM with 128 hidden units to capture low‐frequency temporal features, while the sEMG branch utilized a higher of 512 hidden units to extract fine‐grained temporal dynamics. After non‐linear temporal feature extraction, the fused multimodal features were concatenated and fed into the classification stage with a fully connected network. The model training was performed using the AdamW optimizer with a batch size of 50, a learning rate of 0.001, and a dropout rate of 0.3. The evolution of the loss function and classification accuracy during training is illustrated in Figure S24, with no signs of underfitting or significant overfitting observed. To further validate feature extraction efficacy, fused features were visualized using dimensionality reduction via t‐SNE (Figure 7d).

Upon the completion of training, the Bi‐LSTM model was evaluated on the test dataset. The confusion matrix (Figure 7e) revealed that the model achieved 100% classification accuracy for 23 of the 24 sign types, with an overall accuracy of 99.65% for all 24 sign types. To highlight the advantages of multimodal fusion, experimental comparisons revealed that recognition accuracy dropped to 83.75% and 88.02% when relying solely on strain/pressure or sEMG signals, respectively (Figure S25). This disparity demonstrates that integrating strain/pressure and sEMG signals effectively enhances the system's recognition accuracy. Subsequent system‐level integration experiments confirmed the real‐time translation of sign language into text and synthesized speech, displayed on mobile devices via a WeChat program (Videos S1). As illustrated in Figure 7f, the system successfully recognized the four‐word phrase ‘South China University of Technology’ in real time. Benefiting from the rapid response of the PIG sensor and optimized circuit design, this sign language recognition system achieved near real‐time performance.

## 3 Conclusions

This study successfully developed an ion‐conductive hydrogel with tunable electromechanical properties, offering an effective solution to the challenges of multidimensional and real‐time gesture detection in sign language recognition. The optimized formulation of 0.075 wt.% MBAA and 5 mol L^−1^ AAM exhibited low hysteresiss (recovery ratio of 90.25%), stable strain sensitivity (GF = 2.3 over 0%–550% tensile strain), pressure sensitivity (−0.013 kPa^−^^1^ within 0‐50 kPa), and excellent cyclic stability. A low‐modulus formulation of 0 wt.% MBAA and 5 mol L^−1^ AAM with modulus ≈90 kPa and conductivity = 0.22 S m^−^^1^ was adapted to construct PIG‐Ag electrodes, enabling high‐fidelity sEMG recording with strong resistance to motion artifacts. Incorporation of glycerol/LiCl imparted robust anti‐dehydration capacity, with negligible electromechanical degradation over 14 days. Based on these material advances, a multimodal sign language recognition system was established, comprising two digital gloves (each integrating 12 strain and 5 pressure sensors) and two forearm bands (each incorporating a 10‐channel PIG‐Ag electrode array). Leveraging a dual‐branch Bi‐LSTM multimodal fusion model, the system achieved 99.65% classification accuracy for 24 Chinese sign language gestures. Collectively, these results provide both a reliable materials platform and a robust system‐level framework for next‐generation high‐precision, practical sign language recognition technologies.

## 4 Experimental Section

### 4.1 Materials

Lithium chloride (LiCl), sodium p‐styrenesulfonate (NaSS, 90 wt.%), and glycerol (C_3_H_8_O_3_, 99 wt.%) were procured from Sigma–Aldrich. Lithium Phenyl (2,4,6‐trimethylbenzoyl) Lithium phenyl‐2,4,6‐trimethylbenzoylphosphinate (Lap, 98%) and dimethylaminoethyl acrylate quaternary ammonium salt (DMAEA‐Q, 80 wt.%) were obtained from J&K Chemical Ltd. Acrylamide (AAM, 99 wt.%) and 2,4,6‐trihydroxybenzene‐1,3,5‐tricarbaldehyde compound with benzene‐1,4‐diamine (1:1) (TpPa‐1, 98 wt.%) were purchased from J&K Scientific Co. Ltd. α‐ketoglutaric acid (α‐keto) and *N,N’*‐methylene‐bis‐acrylamide (MBAA) were sourced from Sinopharm Chemical Reagent Co. Ltd. All these reagents, which are of analytical grade, were used as received. For all experiments, deionized water (DI, 18.3 MΩ) was employed.

### 4.2 Synthesis of PNaSS Powder

A homogeneous prepolymer solution was prepared using deionized water as the solvent at 70°C in a water bath. This solution contained 1 m sodium styrene sulphonate (NaSS, 90 wt.%) and 0.05 mol% α‐ketoglutaric acid (*α*‐keto). The prepolymer solution was then rapidly injected into a reaction chamber, whose walls comprised a pair of transparent glass plates fitted with silicone gaskets (10 × 10 × 0.1 cm^3^). Finally, polymerization was conducted at room temperature under UV irradiation (405 nm, 10 W cm^−^^2^) for 10 h to form the pre‐hydrogel. After drying in a 65°C oven for 24 h, the pre‐hydrogel underwent three ball‐milling treatments at 320 rpm. Uniform PNaSS powder was ultimately obtained through a 300‐mesh sieve.

### 4.3 Preparation of PIG Ink

Add TpPa‐1 particles (0.1 wt.%), LiCl (1 mol L^−1^), AAM monomer, and cross‐linking agent MBAA to a 30% glycerol solution. Thoroughly stir the mixture and subject it to ultrasonication for 4 h to ensure uniform dispersion of TpPa‐1 particles and diffusion of monomers into the 1D oriented nanochannels of TpPa‐1. Subsequently, polycationic monomer (DMAEA‐Q), photoinitiator, and polycationic monomer (PNaSS powder) were sequentially added in a 70°C water bath. The molar fraction of the anionic monomer was fixed at 0.5, with the total ionic monomer concentration (*C*_M_) set at 1.15 mol L^−1^. The molar fraction of the photoinitiator is maintained at 0.1 mol% relative to *C*_M_. After thorough heating and stirring, the PIG ink was prepared.

### 4.4 3D printing of PIG

PIG's 3D printing was accomplished using a microelectronic printing platform (Power Square's MP series). Prior to printing, degassed PIG ink was loaded into syringes and extruded through the nozzle under pneumatic pressure. To achieve high‐resolution patterning, parameters such as air pressure, nozzle diameter, nozzle‐to‐substrate distance, and nozzle speed were optimized. Following printing, complete gel polymerization was achieved through exposure to UV light (405 nm, 10 W cm^−^^2^) for 2 h.

## Ethical Declaration

Ethics approval and informed consent were obtained from all participants and the protocol approved by the Maoming People's Hospital Department of Ethics Committee (PJ2020MI‐K179‐ 01). The study was carried out under the Declaration of Helsinki.

## Conflicts of Interest

The authors declare no conflicts of interest.

## Supporting information




**Supporting File 1**: advs73938‐sup‐0001‐SuppMat.pdf.

**Supporting File 2**: advs73938‐sup‐0002‐VideoS1.mp4.

## Data Availability

The data that support the findings of this study are available from the corresponding author upon reasonable request.
