# Supplementary material for: 3D‐Printed Ion‐Conductive Hydrogels with Tunable Mechanical–Electrical Properties for Multimodal Sign Language Recognition
Source: Adv Sci (Weinh). 2026 Jan 20;13(18):e20586. doi: 10.1002/advs.202520586 (PMC13042474; doi:10.1002/advs.202520586)
Supplement: Supplementary file 1 — Supporting File 1: advs73938‐sup‐0001‐SuppMat.pdf. [file ADVS-13-e20586-s001.pdf]

Supporting Information

3D-Printed ion-conductive hydrogels with tunable mechanical–electrical properties for multimodal sign language recognition

Quan Hu[#], *Longya Xiao*[#], *Peiqi Zhang, Qiurui Zhang, Guangsen Liu, Xinglin Qin, Zhuhui Yin, Xian Li, Yuling Wang, Hongjie Jiang**

S1. Supplementary Methods

S1.1. Tensile Tests of PIG

All mechanical experiments were conducted using a universal testing machine (Model HZ-1004A, China) equipped with a 5 kN load cell. For the monotonic tensile test, specimens were prepared in a dumbbell shape with a gauge length of 12 mm, width of 2 mm, and thickness of 1 mm. The tensile rate was 50 mm·min^{-1} (strain rate 7% s^{-1}). Actual test dimensions were measured precisely using vernier calipers. Specimen ends were fully clamped to prevent shoulder stretching, with sandpaper applied to prevent slippage between specimen and grips. No slippage was observed throughout testing, with all specimens fracturing within the mid-linear region. Young's modulus (E) was calculated from the slope of the initial linear region (strain ≤ 10%) of the stress-strain curve. Stress was calculated by dividing the measured load by the specimen's cross-sectional area. Strain values were determined by dividing the measured displacement by the initial gauge length. The tension work (W_t) absorbed by the specimen was calculated by integrating the area under the stress-strain curve, as per the following formula:

$$W_{\mathrm{t}} = \int_{0}^{\varepsilon_b} \sigma \, d\varepsilon \qquad \text{(e1)}$$

, where σ and ε denote stress and strain respectively, and ε_b represents the strain value at

specimen fracture. Each specimen shall be tested at least three times, with the mean value and standard deviation calculated; the standard deviation shall be represented as error bars.

S1.2. Compression Tests of PIG

For monotonic compression tests, specimens was prepared in a cylindrical form with a diameter of 10 mm and a height of 3 mm. Monotonic compression testing was conducted along the height of the cylinder at a compression rate of 3 mm·min^{-1} (strain rate 1.7% s^{-1}; gauge length 3 mm). Actual test dimensions were measured precisely using a vernier caliper. No slippage was observed throughout the test. The compressive elastic modulus (E_c) was calculated from the slope of the initial linear region (strain ≤ 10%) of the stress-strain curve. The compression work (W_c) absorbed by the specimen at different strains is obtained by integrating the stress-strain curve, as per the following formula:

$$W_c = \int_0^{\varepsilon} \sigma \, d\varepsilon \quad \text{(e2)}$$

, where σ and ε denote stress and strain respectively. Each specimen shall be tested at least three times, with the mean value and standard deviation calculated; the standard deviation shall be represented as error bars.

S1.3. Tearing Tests of PIG

Trouser tearing tests of the hydrogel samples were performed at ambient temperature to characterize the tearing energy. The samples were cut into rectangular shape (L (50 mm) × w (10 mm) × t (≈1 mm)) with a 20 mm notch in the middle, as illustrated in Figure S14a. The two arms of the samples were clamped, and the upper arm was pulled at a constant velocity of (100 mm min^{-1}) until the crack advanced through the entire sample, while the tensile force was recorded. The tearing energy, T, was calculated as follows:

$$T = \frac{2\bar{F}}{t} \tag{e3}$$

, where $\bar{F}$ and t are the average tearing force to advance the crack and the sample thickness, respectively.

S1.4. Adhesion test of PIG

The interfacial toughness of PIG in contact with pig skin was evaluated using the 180-degree peel test method specified in ASTM F2256. Adhesion strength was determined by calculating the average force at stable peel conditions and dividing by the adhesion width. Each specimen measured 50 mm in length, 15 mm in width, and 2 mm in thickness. To prevent hydrogel samples from stretching during adhesion testing, cyanoacrylate adhesive was employed to bond them to polyethylene terephthalate (PET) film. Each sample underwent a 60-second application of a 5N load prior to testing. All tests were conducted using a universal testing machine at a constant tensile speed of 100 $mm \cdot min^{-1}$.

S1.5. Electrical characterizations of PIG

The electrical properties of the PIG were measured using a LCR meter (E4980AL, KEYSIGHT) and a digital multimeter (34465A, KEYSIGHT). The sample resistance (R) was calculated under an excitation frequency of 1000 Hz. The ionic conductivity (σ) was calculated using the formula $\sigma = L/(R \cdot S)$, where L represents the distance between the two electrodes and S denotes the cross-sectional area of the sample. AC impedance was assessed using an electrochemical analyzer (CHI627D, Shanghai Chenhua, China) equipped with a three-electrode system. During measurement, the reference electrode and counter electrode were positioned 3 cm and 6 cm respectively from the working electrode. The frequency scan range was set from 1 to 1000 Hz to encompass the primary spectral range of the sEMG signal. The

signal-to-noise ratio (SNR) of the sEMG signal was calculated as follows:

$$SNR(dB) = 20 \times log \frac{\sqrt{\sum_{k=1}^{N} V_{singal(k)}^2}}{\sqrt{\sum_{k=1}^{N} V_{noise(k)}^2}} \tag{e4}$$

S2. Viscoelastic Modelling of PIG

The Rheological property of PIG uses the viscoelastic model proposed by Creton et al. to understand the viscoelastic and elastic contributions in viscoelastic soft materials.[1-3] The model consists of a parallel combination of the Upper Convected Maxwell (UCM) model describing viscoelastic flow and Gent strain hardening model for entropic elasticity of finite extensibility of polymer chains. The nominal stress (σ_N) can be expressed by the sum of the UCM element and Gent element contributions as:

$$\sigma_{N}(\lambda) = \sigma_{N,\ v}(\lambda) + \sigma_{N,\ e}(\lambda) \tag{e5}$$

, where the details of the individual parts are:

$$\sigma_{N,\ v}(\lambda) = \left(\frac{2G_v D_e}{1-2D_e}\left(1-\exp\left(-\frac{1-2D_e}{D_e}(\lambda-1)\right)\right) + \frac{G_v D_e}{1+D_e}\left(1-\exp\left(-\frac{1+D_e}{D_e}(\lambda-1)\right)\right)\right)\cdot\lambda^{-1} \tag{e6}$$

for viscoelastic stress, and

$$\sigma_{N,\ e}(\lambda) = \left(\frac{G_e}{1-\frac{\lambda^2+2\lambda^{-1}-3}{J_m}}(\lambda^2-\lambda^{-1})\right)\cdot\lambda^{-1} \tag{e7}$$

for entropic elastic stress. G_v and G_e are the initial shear modulus of the viscoelastic part (contributed by relatively weak bonds' rupture) and the elastic part (contributed by relatively strong physical bonds and covalent bonds). De is the Deborah number and J_m represents the

maximum allowable value of the first strain variable, indicating the theoretical finite extensibility of the network chain. Under uniaxial tensile conditions, it can be expressed as:

$$J_{\mathrm{m}} = \lambda_{\mathrm{m}}^{2} + 2\lambda_{\mathrm{m}}^{-1} - 3 \tag{e8}$$

, where λ_m represents the theoretical maximum elongation ratio of the polymer chain. $\sigma_N(\lambda)$ is determined by G_v, G_e, D_e, and J_m. Since Young's modulus $E \approx 3(G_v + G_e)$, tensile data can be fitted using this model, which contains three independent parameters and E (obtained from tensile testing).

S3. Supplementary Figures

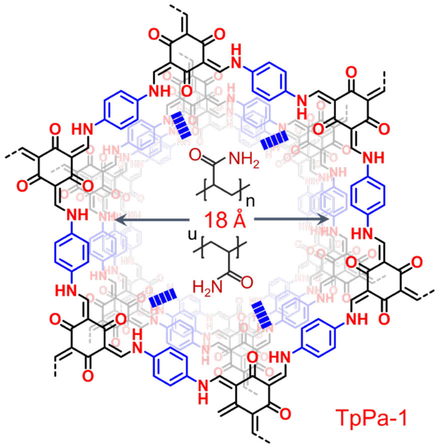

Figure S1. Molecular structure of TpPa-1.

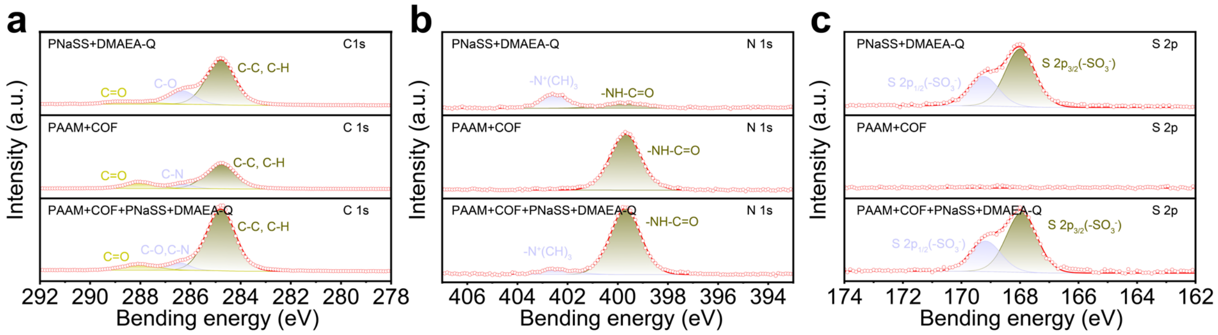

Figure S2. XPS spectra of PIG (PAAM+COF+PNaSS+DMAEA-Q), PAAM+COF, and PNaSS+DMAEA-Q.

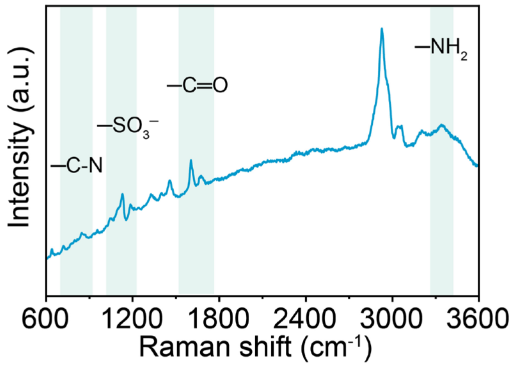

Figure S3. Raman spectrum of PIG, indicating the successful synthesis of PIG (PAAM+COF+PNaSS+DMAEA-Q).

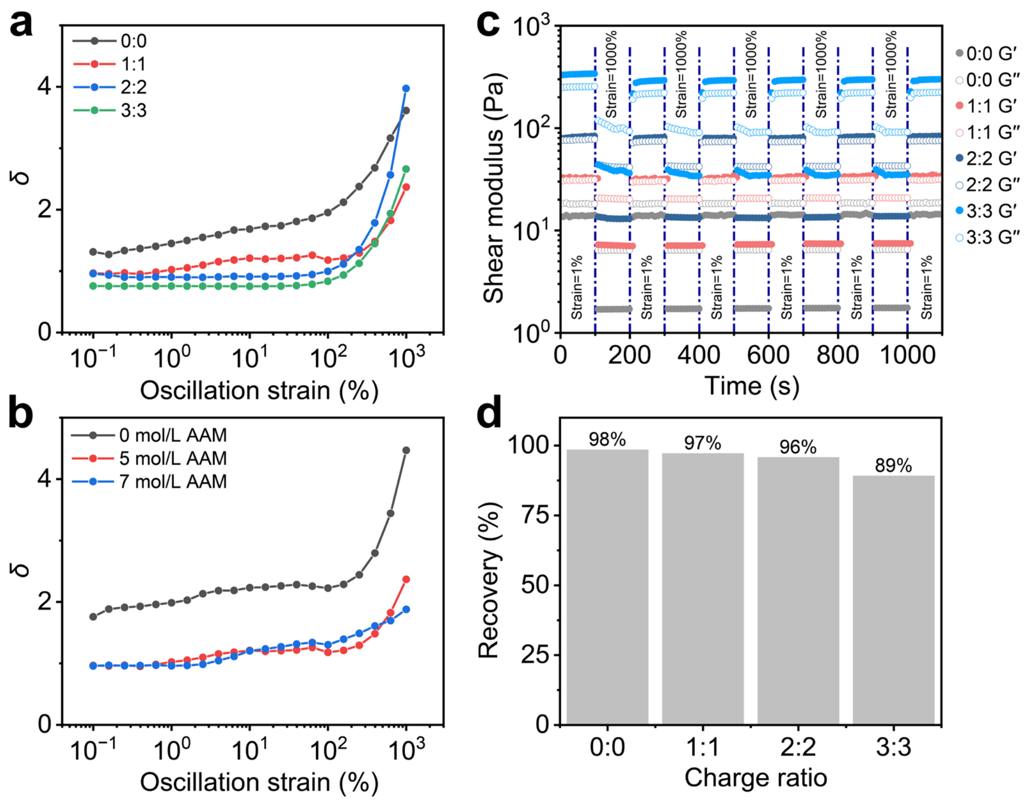

Figure S4. Rheological testing of PIG ink. The variation of δ (where $\delta = G''/G'$, with G'' being the loss modulus and G' the storage modulus) versus cyclic strain under different (a) charge ratios and (b) AAM concentrations. When $\delta > 1$, the ink exhibited liquid behavior; when $\delta < 1$, it demonstrated solid behavior. The results indicate that when the AAM concentration is 5 mol L^{-1} and the charge ratio is 1:1, the PIG ink undergoes a liquid-solid phase transition. (**c**) Time-dependent analyses of G' and G'' alternately applying small (10%) and large (1000%) oscillatory angular strain by 5 cycles with varying charge ratios from 0:0 to 3:3. (**d**) The recovery rate of PIG inks, obtained by dividing the final 100-second $G'_{\text{ultimately}}$ value with the initial 100-second G'_{initiall} value, showing a significant decrease when increasing charge ratio from 0:0 to 3:3.

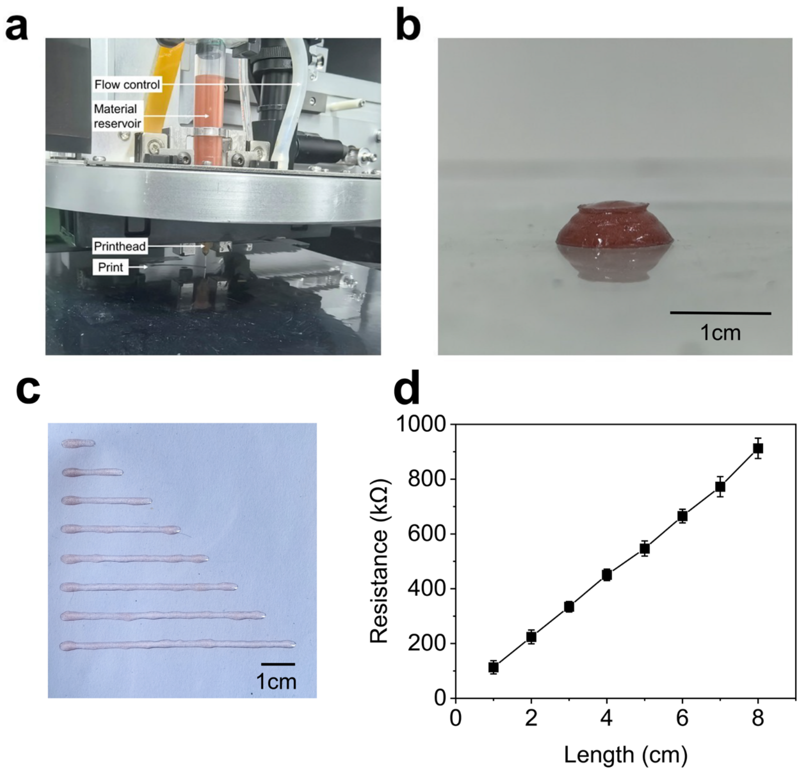

Figure S5. Demonstration of the 3D printing performance of the PIG ink. (**a-b**) Photographs showing the printing of the PIG ink into a 3D hemispherical structure via a commercial inkjet printer. (**c-d**) Eight gel strips with lengths ranging from 1 cm to 8 cm, demonstrating a linear relationship between electrical resistances and lengths, confirming the uniform conductivity and printing precision.

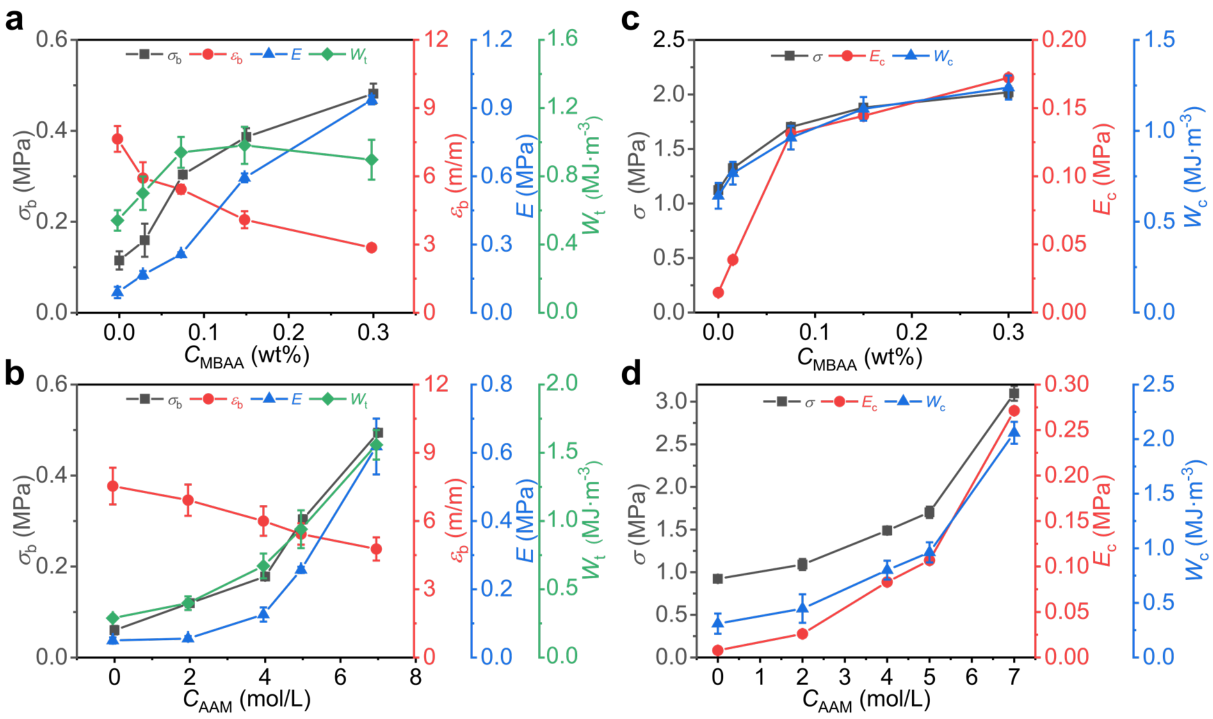

Figure S6. Mechanical properties of PIG prepared with varying concentrations of MBAA and AAM. (**a,b**) Monotonic tensile tests showing variations in fracture strength (σ_b), fracture strain (ε_b), Young's modulus (E), and tensile work (W_t) of PIG as a function of MBAA and AAM concentrations. (**c,d**) Monotonic compression tests showing the dependence of 80% compressive strength (σ), compressive elastic modulus (E_c), and compressive work (W_c) on MBAA and AAM concentrations.

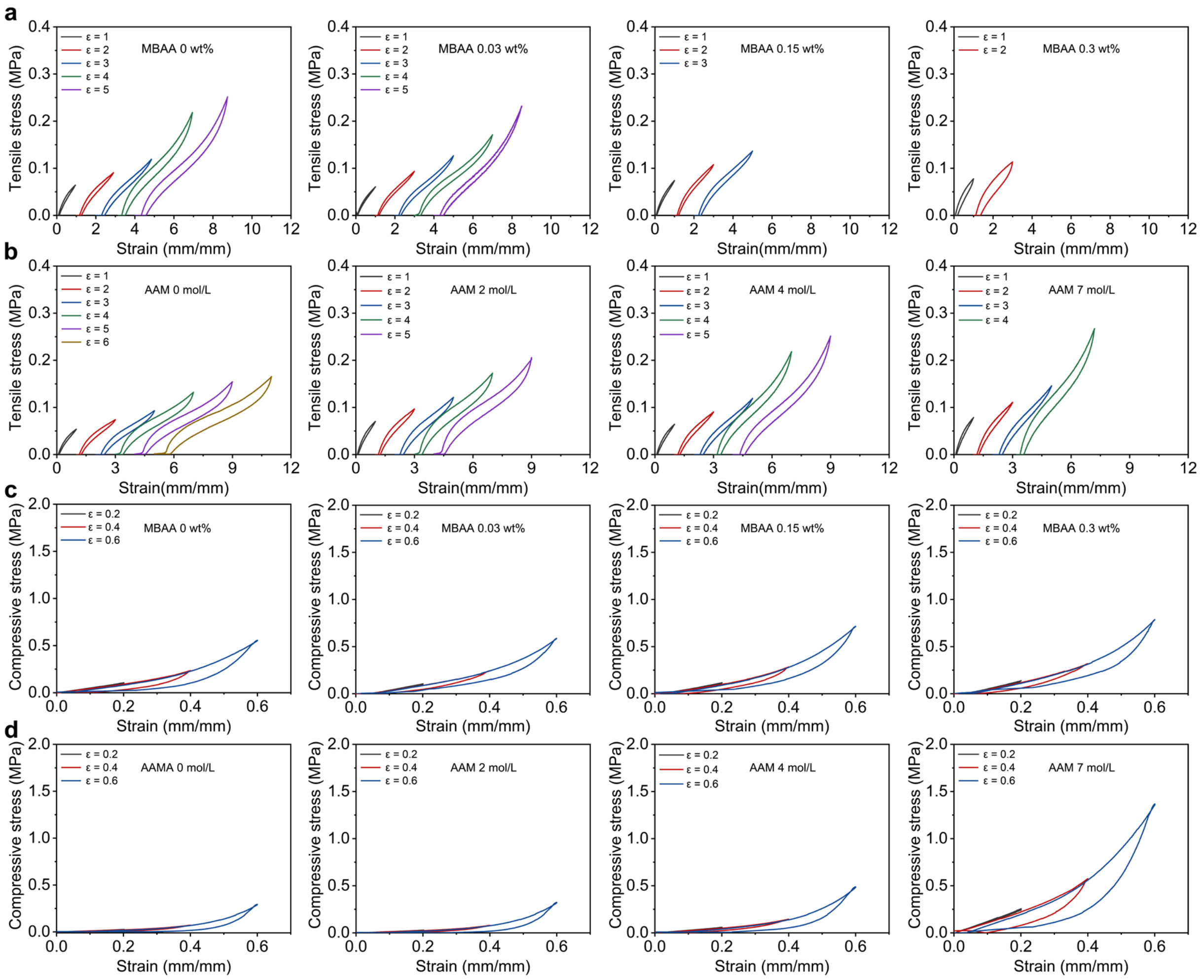

Figure S7. Load-unload stress-strain behavior of PIGs with varying compositions and strain levels. (**a,b**) Tensile load-unload stress-strain curves of PIGs prepared with different concentrations of MBAA and AAM under various tensile strains. (**c,d**) Compressive load-unload stress-strain curves of PIGs with different MBAA and AAM concentrations under varying compressive strains.

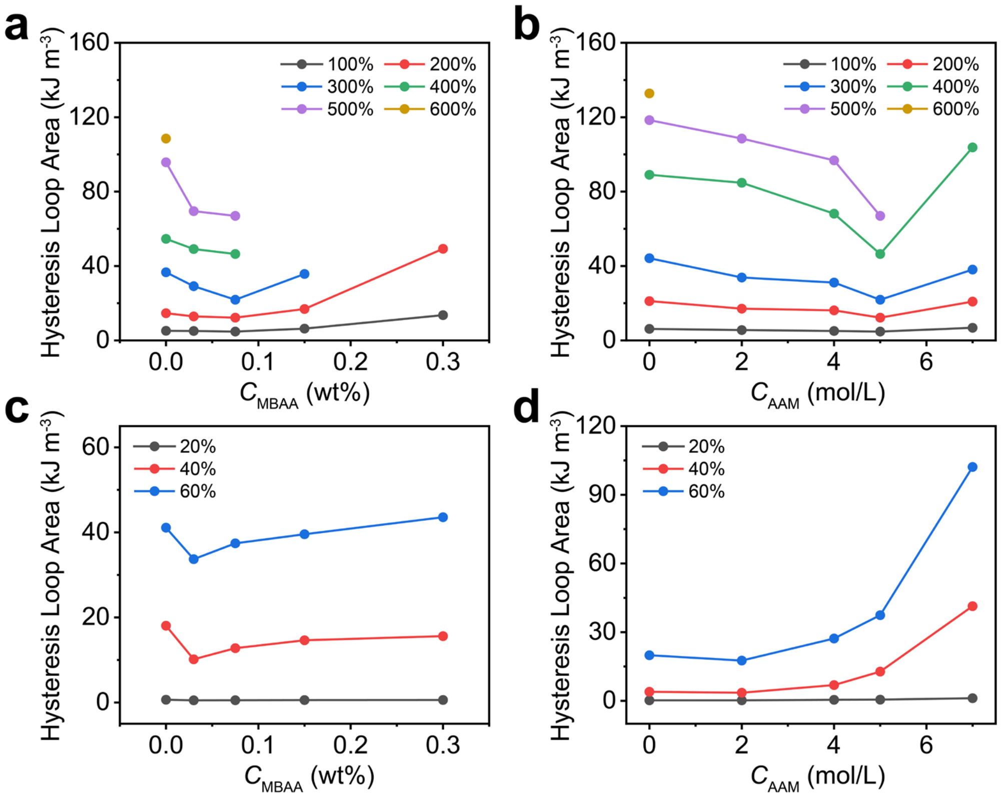

Figure S8. Hysteresis behavior of PIGs with different compositions under varying tensile and compressive strains. At tensile strain from 100% to 600% (**a,b**) or compressive strain from 20% to 60% (**c,d**), the hysteresis loop area first decreases and then increases with increasing MBAA or AAM content.

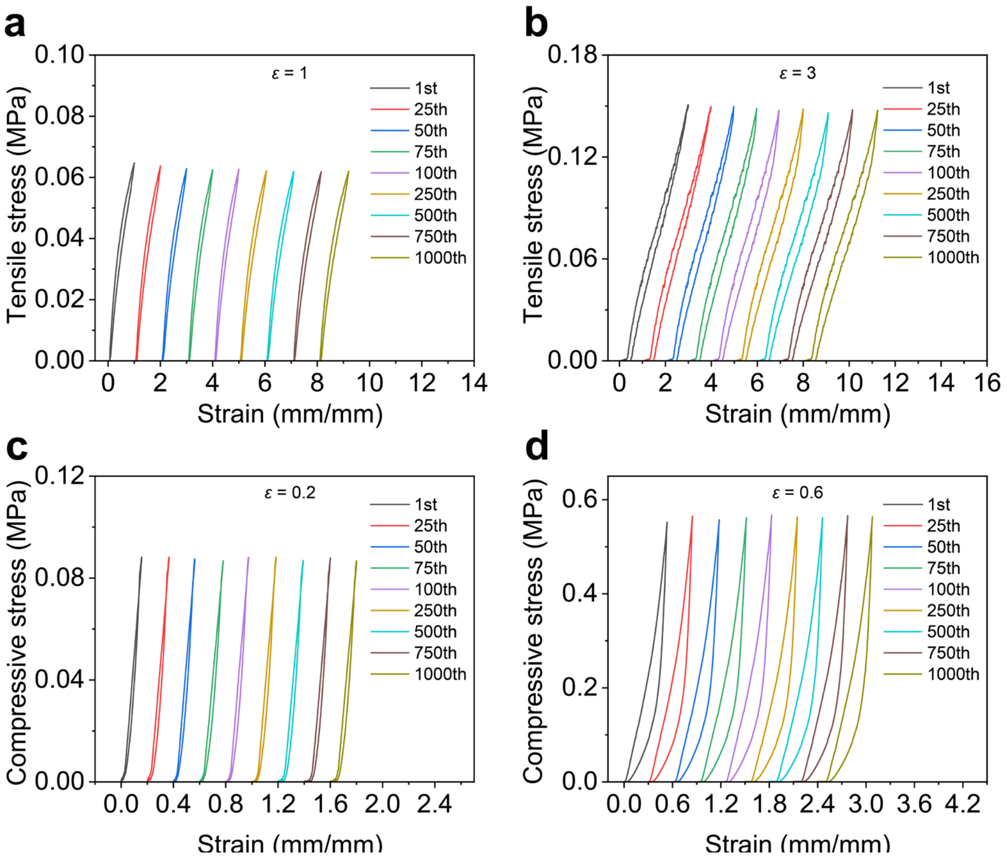

Figure S9. Cyclic tensile and compressive behaviors of $PIG_{0.075-5}$ under repeated loading. (**a,b**) Tensile stress-strain behaviors of $PIG_{0.075-5}$ during 1000 consecutive loading-unloading cycles at tensile strains of ε = 1 and 3. The hysteresis loop area and residual strain showed no significant changes. (**c,d**) Compressive stress-strain curves of $PIG_{0.075-5}$ recorded over 1000 consecutive cyclic loading-unloading cycles under compressive strains of ε = 0.2 and 0.6. The hysteresis loop area and residual strain showed no significant changes.

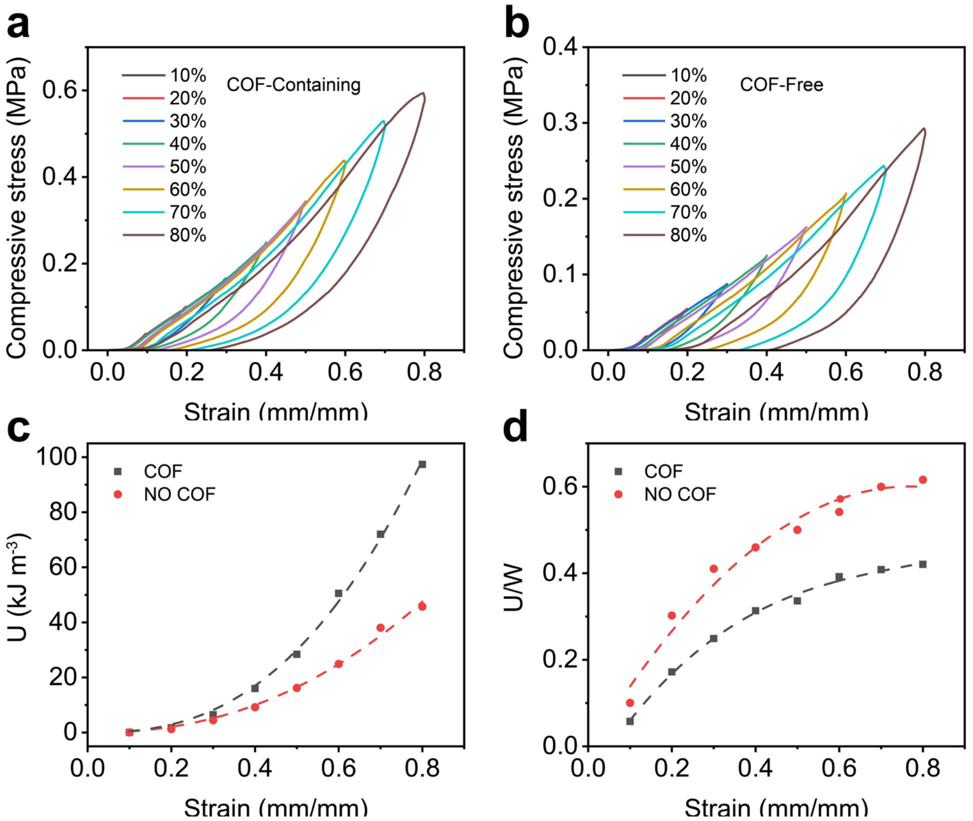

Figure S10. Effect of COF on energy dissipation during escalating compression upon $PIG_{0.075-5}$. (**a,b**) Continuous and cyclic compressive stress-strain curves for PIGs with and without COF embedding. (**c**) Comparison of dissipated energy (U), and (**d**) the ratio of dissipated energy to total tensile work (U/W) between $PIG_{0.075-5}$ with and without COF embedding. The results show that introducing COF increases the U value while decreasing the U/W ratio, indicating that COF enhances the material's effective energy dissipation and hysteresis resistance.

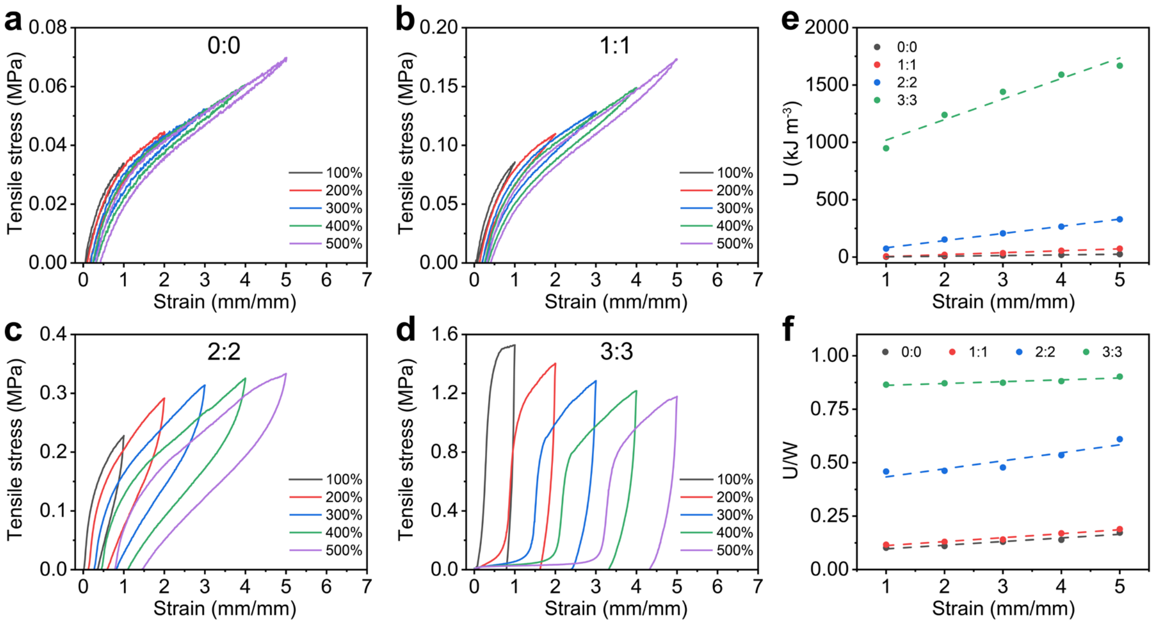

Figure S11. The effect of charge ratio between PNaSS and DMAEA-Q on energy dissipation during incremental stretching of $PIG_{0.075\text{-}5}$. (**a-d**) Continuous cyclic tensile stress-strain curves of $PIG_{0.075\text{-}5}$ materials with different charge ratios. (**e**) Comparison of dissipated energy (U) among $PIG_{0.075\text{-}5}$ materials with varying charge ratios; (**f**) Comparison of the dissipated energy-to-total tensile work ratio (U/W) for $PIG_{0.075\text{-}5}$ materials with different charge ratios. Results show that both the U value and U/W ratio increase with rising charge ratio, suggesting that increased crosslinking density enhances the gel's energy dissipation while reducing its hysteresis resistance.

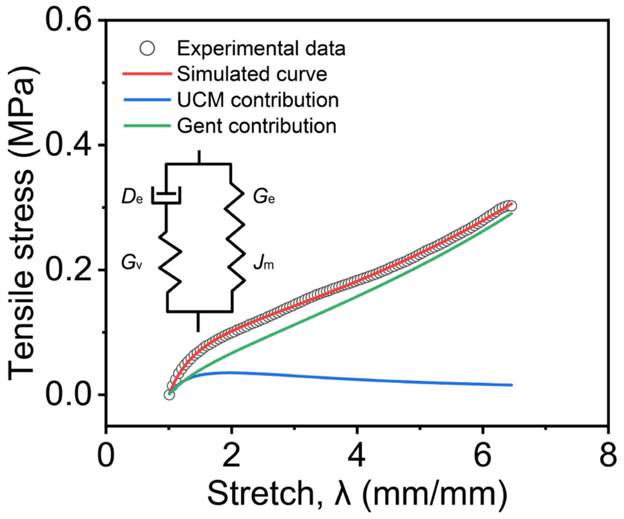

Figure S12. Viscoelastic modelling of $PIG_{0.075\text{-}5}$, including its tensile behavior and the simulated curve showing contributions from the viscoelastic part (UCM, G_v) and elastic part (Gent, G_e), based on the viscoelastic model. The insert shows a sketch of the viscoelastic model. The fitting result confirms that this combined model effectively captures the tensile behavior of the gels in our work. This model allows for the separation of the shear modulus into its viscoelastic (G_v) and elastic (G_e) components.

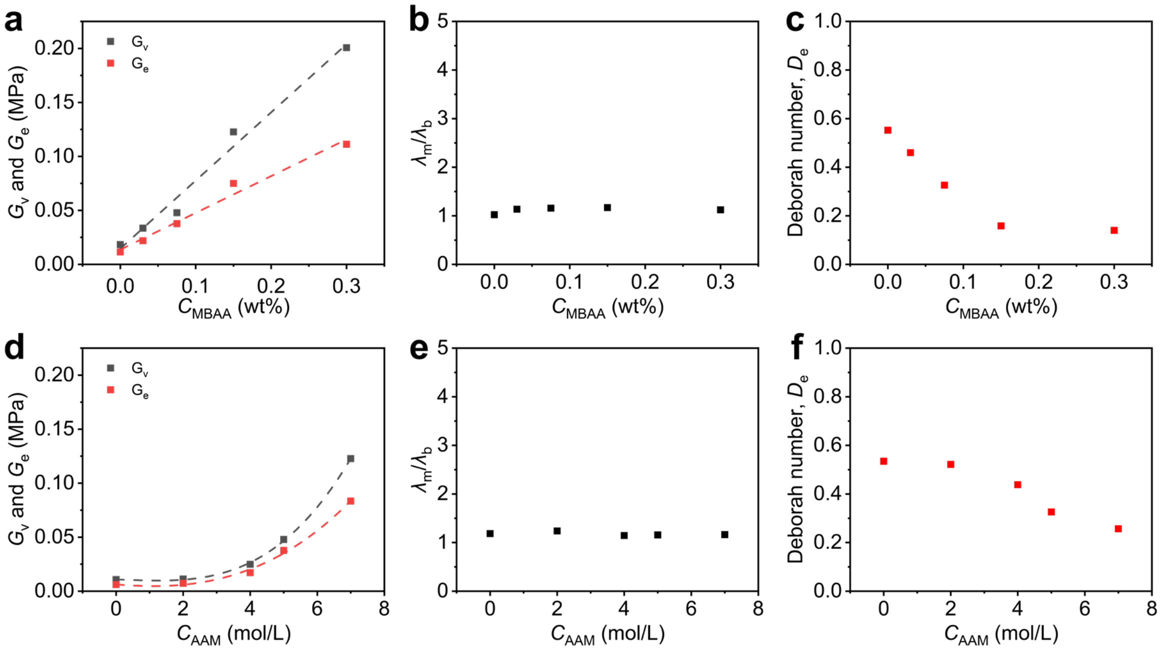

Figure S13. Viscoelastic model fitting parameters for PIGs at different MBAA and AAM concentrations. (**a,d**) Viscoelastic (G_v) and elastic (G_e) components of the initial shear modulus increase with increasing MBAA and AAM concentrations. (**b,e**) The λ_m/λ_b ratio remains approximately constant around 1 as MBAA and AAM concentrations increase, where λ_m represents the limiting stretch ratio of polymer chains in the Gent model and λ_b is the experimental stretch ratio at breaking (i.e., $\varepsilon_b + 1$) based on tensile data. (**c,f**) The Deborah number (D_e) of the viscoelastic component decreases gradually with increasing MBAA and AAM concentrations, which is proportional to the relaxation time of the viscoelastic parts.

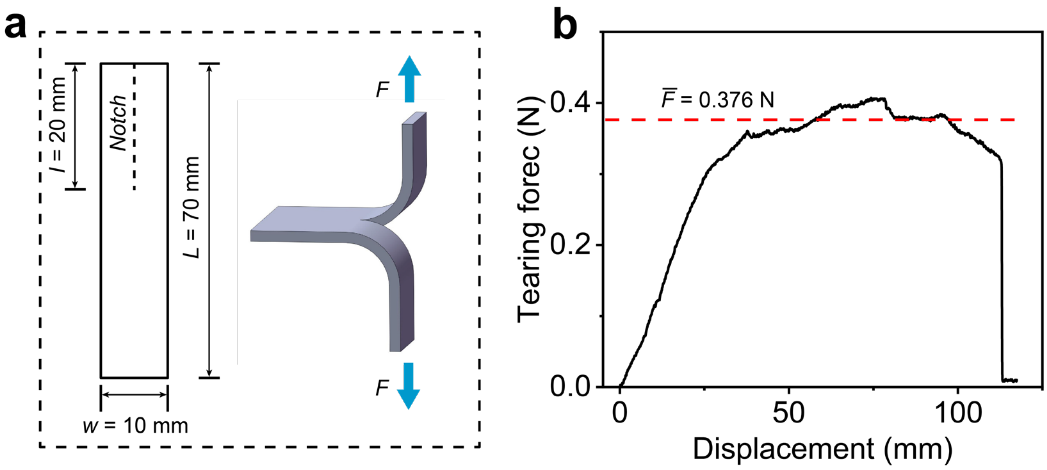

Figure S14. (**a**) Schematic illustration of the sample geometry and methodology used in the trouser tearing test. (**b**) Representative tearing force–displacement curve of $PIG_{0.075\text{-}5}$, with the average steady-state tearing force ($\bar{F}$) obtained for the calculation of tearing energy.

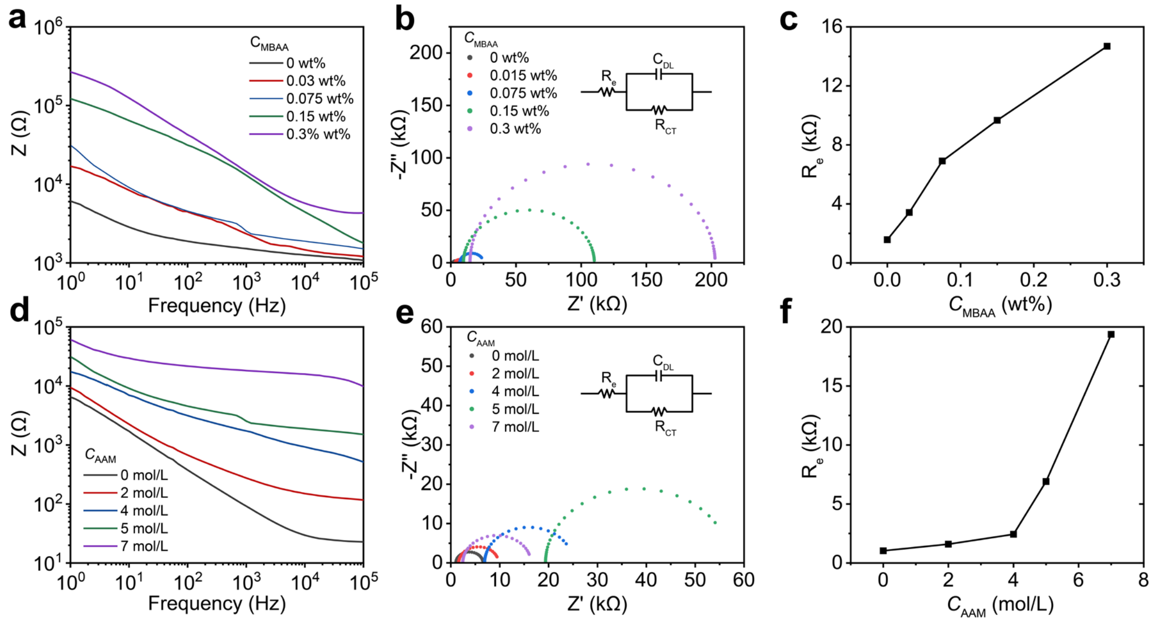

Figure S15. Electrochemical impedance characterizations of PIGs with different compositions. (**a,d**) Impedance spectra of PIG across the excitation frequency range from 1 Hz to 10^5 Hz, showing a decrease in impedance with increasing frequency, and an increase in impedance with higher MBAA and AAM contents. This may result from the increased crosslinking density associated with higher MBAA and AAM. (**b,e**) Nyquist plot of PIG as a function of MBAA and AAM content, with the inset showing the corresponding electrical equivalent model. (**c,f**) EIS fitting showing that the bulk resistance (R_e) of the gel increases with higher MBAA and AAM content. C_{DL} and R_{CT} represent the double-layer capacitance and charge transfer resistance of the gel and electrode, respectively.

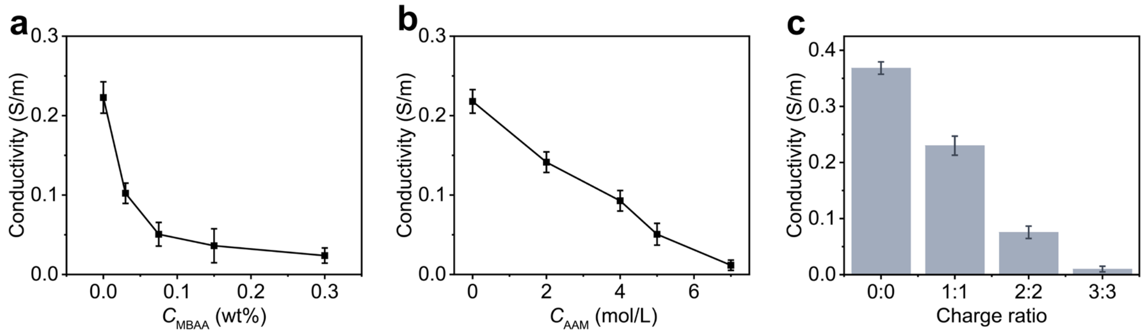

Figure S16. Effect of composition on the electrical conductivity of PIG. (**a**) When the AAM is fixed at 5 mol L^{-1} and the charge ratio is 1:1, the conductivity of PIG decreases with the increase of MBAA content. (**b**) When MBAA is fixed at 0.075 wt% and the charge ratio is 1:1, the conductivity of PIG decreases with the increase of AAM content. (**c**) The conductivity of $PIG_{0\text{-}5}$ decreases with the increase of the charge ratio.

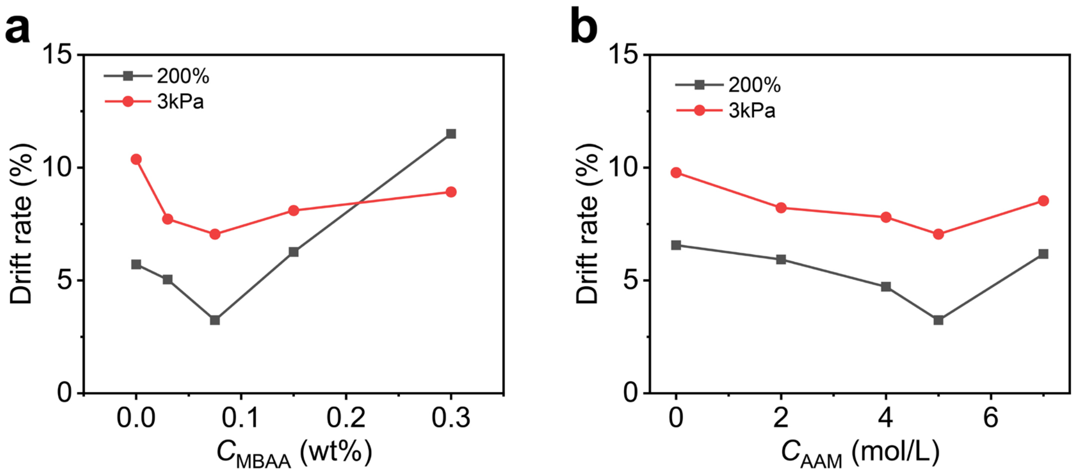

Figure S17. Signal drift in PIG-based strain and pressure sensors after undergoing 500 cycles of 200% tensile stress and 5000 cycles of 3 kPa pressure exhibits a trend of initially decreasing and then increasing with rising MBAA and AAM content. Using formulation of $PIG_{0.075\text{-}5}$, both sensors exhibited the lowest signal drift rate, consistent with the composition dependent recovery rate pattern observed.

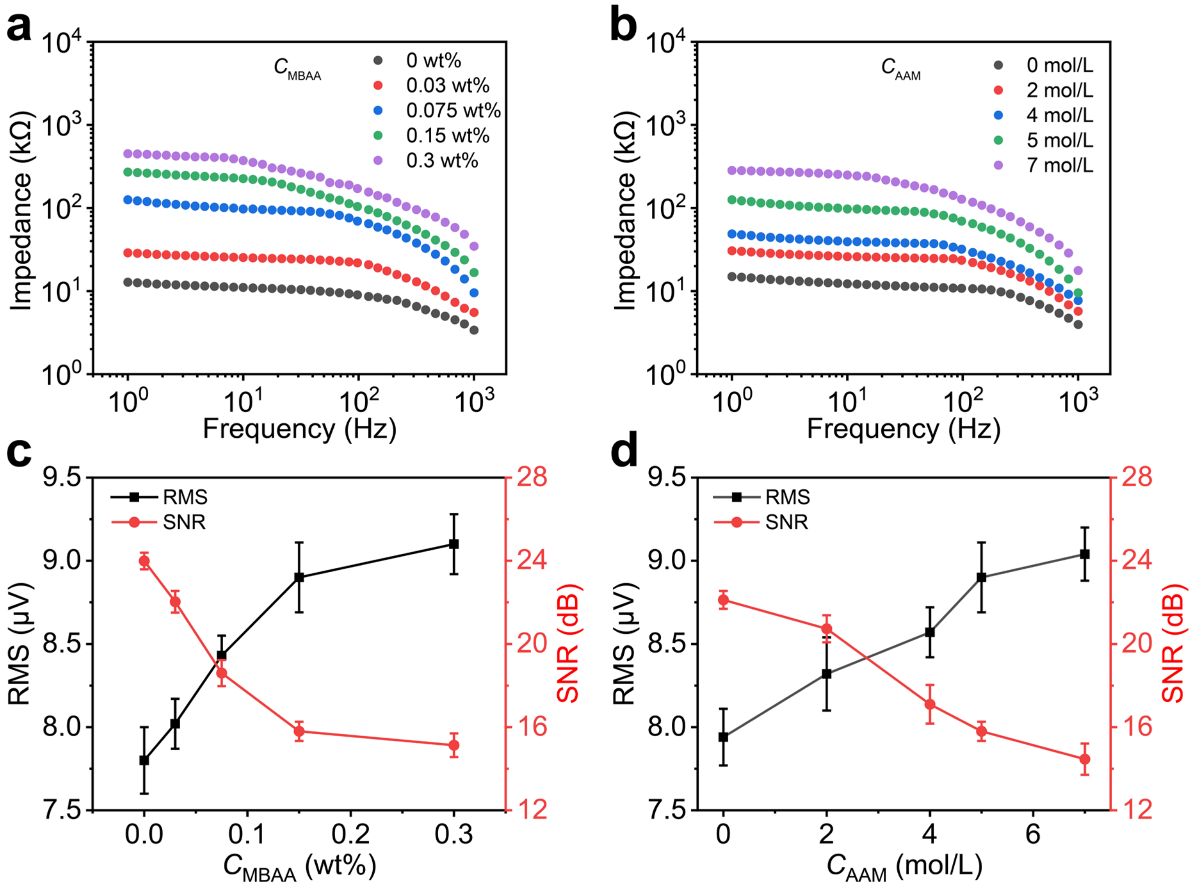

Figure S18. Differences in sEMG signal detection among PIGs with different compositions. (**a,b**) PIG-skin interface impedance increases with rising MBAA and AAM content. (**c,d**) For PIG-Ag electrodes with different compositions, RMS values increase while SNR decreases during 50% MVC dynamic interference testing as MBAA and AAM content rises.

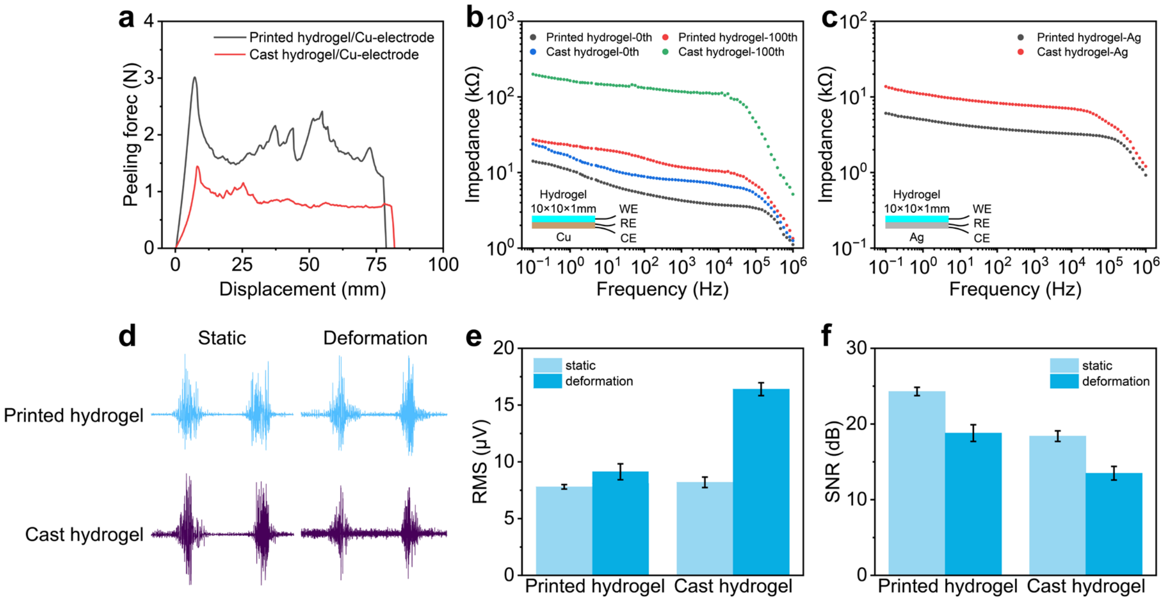

Figure S19. Electromechanical performance comparison between printed and cast hydrogels. (**a**) The printed $PIG_{0.075\text{-}5}$ with Cu electrode exhibits higher adhesion force compared to the cast hydrogel with Cu electrode. (**b**) Printed $PIG_{0.075\text{-}5}$ and (**c**) $PIG_{0\text{-}5}$ with Cu/Ag electrode demonstrates lower contact impedance, both before and after 100 consecutive 90° bends, across the excitation frequency range from 10^{-1} Hz to 10^6 Hz, compared to the cast hydrogel with Cu/Ag electrode. (**e-f**) Static and dynamic sEMG characterizations using printed and cast hydrogel electrodes at 50% MVC, showing similar RMS values for static measurements with printed hydrogel electrodes, but a much higher increase in dynamic movement using cast hydrogel electrodes. SNR also shows better performance with printed hydrogel electrodes.

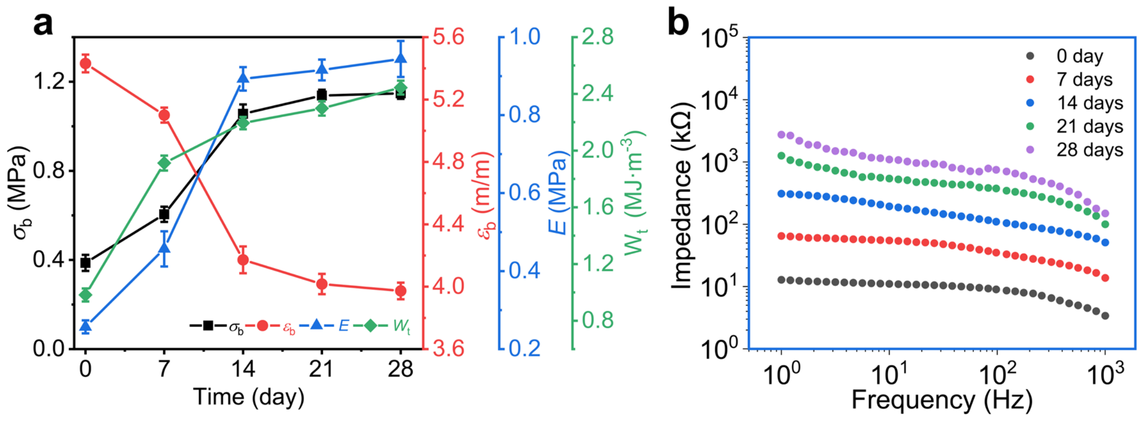

Figure S20. Environmental aging characterizations of PIG stored under ambient conditions for 28-day. (**a**) The fracture strength (σ_b), fracture strain (ε_b), Young's modulus (E), and tensile work (W_t) of the $PIG_{0.075-5}$ were evaluated at 7-day intervals to assess the influence of storage duration on its mechanical performance. (**b**) Time-dependent changes in the interfacial impedance between the PIG_{0-5}-Ag electrode and the skin.

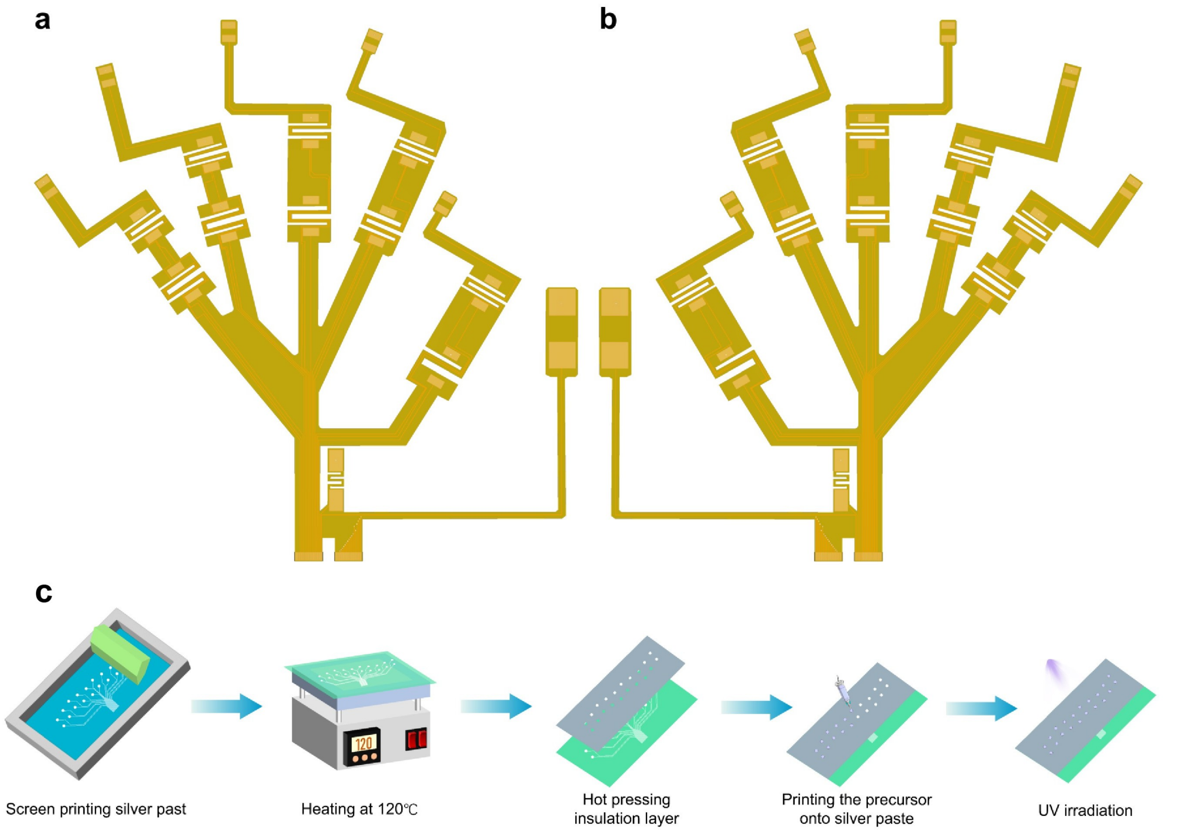

Figure S21. Schematic diagram of the conductive electrode structure for strain/pressure and sEMG sensing. (**a,b**) Photographs of the glove-patterned flexible printed circuit board (FPCB) with serpentine interconnects linking PIG-based strain/pressure sensors to the readout circuit. (**c**) Schematic illustration of a 10-channel differential sEMG electrode array fabricated using screen printing and layer-by-layer imprinting techniques.

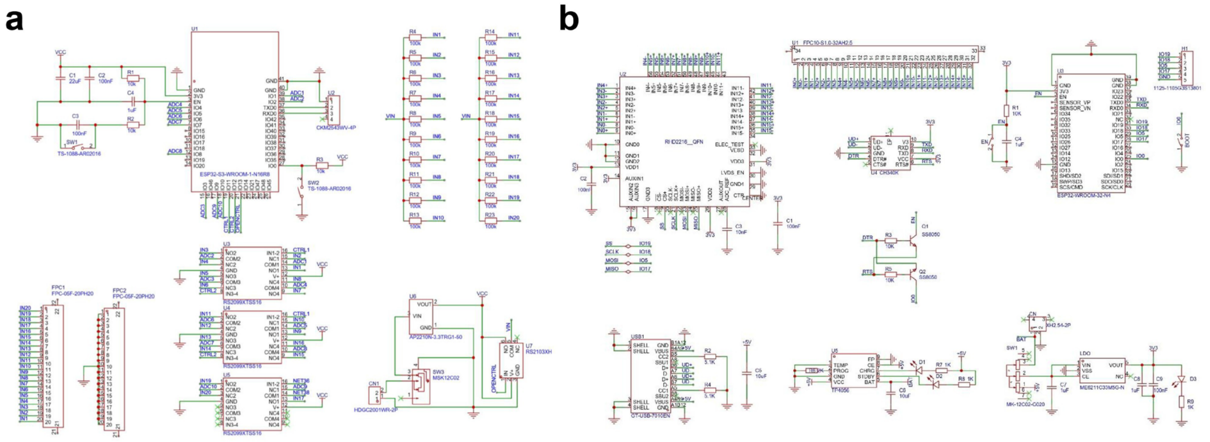

Figure S22. Signal acquisition circuit schematic diagram. Schematic layout of the readout circuit (**a**) for strain and pressure sensors, and (**b**) for sEMG sensors.

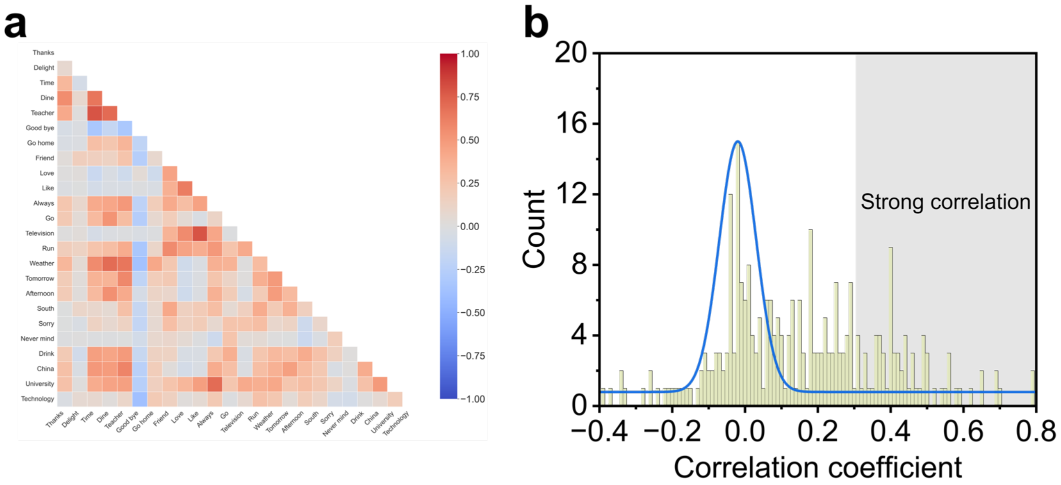

Figure S23. Correlation analysis of multimodal signals across 24 sign language words. (**a**) Correlation coefficient matrix illustrating pairwise signal similarity among 24 gesture categories. (**b**) Distribution curve of correlation coefficients, highlighting inter-gesture similarity and potential overlap in signal patterns.

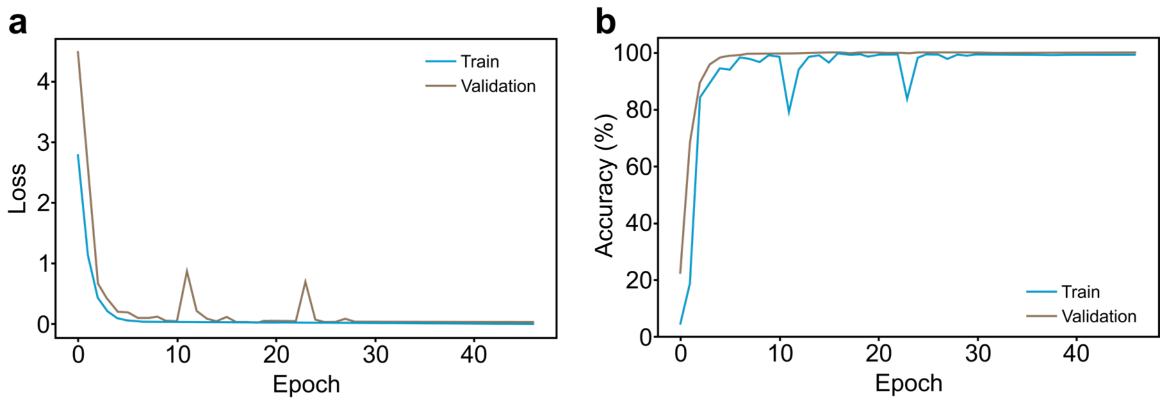

Figure S24. Evolution of model loss and accuracy during training. (**a**) Variation of the model's loss value with each training batch, showing progressive convergence as training proceeds. (**b**) Variation of model accuracy with each training batch, illustrating the improvement in predictive performance.

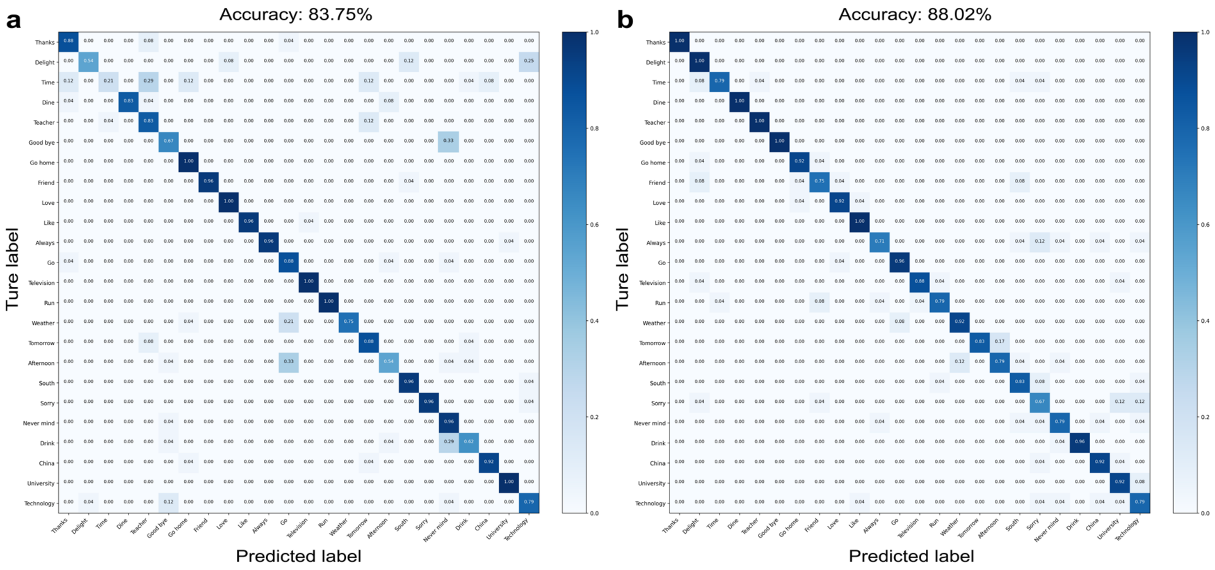

Figure S25. Confusion matrix diagram for recognizing 24 sign language gestures using only (**a**) strain/pressure or (**b**) sEMG as a single modality.

S4. Supplementary Tables

Table S1. EIS fitting results for gel impedance as a function of MBAA and AAM.

C_{MBAA} (wt%)	R_e(kΩ)	C_{DL}(μF)	R_{CT}(kΩ)
0	1.569	7.74	3.668
0.015	3.419	5.65	5.887
0.075	6.904	1.83	18.11
0.15	9.666	0.0337	100.5
0.3	14.682	0.0189	188.1
C_{AAM} (mol L^{-1})	R_e(kΩ)	C_{DL}(μF)	R_{CT}(kΩ)
0	1.035	3.8	5.535
2	1.596	2.91	8.148
4	2.431	2.55	13.94
5	6.904	1.83	18.11
7	19.37	1.25	37.73

S5. Supplementary Movie

Movie S1. A sign language recognition system built using PIG sensors was employed to recognize 24 distinct sign languages. The results demonstrated that the system effectively identified different sign languages.

Supplementary References

[1] T. L. Sun, F. Luo, T. Kurokawa, S. N. Karobi, T. Nakajima, J. P. Gong, *Soft Matter* **2015**, 11, 9355.

[2] L. Xiao, Y. Huang, S. Qian, S. Long, H. Jiang, P. Rao, X. Li, *Chem. Eng. J.* **2025**, 507, 160657.

[3] A. B. Ihsan, T. L. Sun, T. Kurokawa, S. N. Karobi, T. Nakajima, T. Nonoyama, C. K. Roy, F. Luo, J. P. Gong, *Macromolecules* **2016**, 49, 4245.
